# Advances in HIV-1 Vaccine Development

**DOI:** 10.3390/v10040167

**Published:** 2018-04-01

**Authors:** Yong Gao, Paul F. McKay, Jamie F. S. Mann

**Affiliations:** 1 Department of Microbiology and Immunology, University of Western Ontario, London, ON, N6A 5C1, Canada; ygao387@uwo.ca; 2 Imperial College London, Department of Infectious Diseases, Division of Medicine, Norfolk Place, London, W2 1PG, UK; p.mckay@imperial.ac.uk

**Keywords:** HIV-1, vaccine, antibodies, immunogen, neutralization

## Abstract

An efficacious HIV-1 vaccine is regarded as the best way to halt the ongoing HIV-1 epidemic. However, despite significant efforts to develop a safe and effective vaccine, the modestly protective RV144 trial remains the only efficacy trial to provide some level of protection against HIV-1 acquisition. This review will outline the history of HIV vaccine development, novel technologies being applied to HIV vaccinology and immunogen design, as well as the studies that are ongoing to advance our understanding of vaccine-induced immune correlates of protection.

## 1. Introduction

The lentiviral retrovirus, Human Immunodeficiency Virus type 1 (HIV-1), is the etiological agent behind the global Acquired Immunodeficiency Syndrome (AIDS) epidemic. Since the AIDS epidemic was first identified in the early 1980s, approximately 70 million individuals have become infected, resulting in 35 million deaths. The introduction of combination antiretroviral therapy (cART) has dramatically altered the epidemic landscape and has been responsible for the 48% decline in AIDS related deaths between 2005 and 2016 [1]. Despite this remarkable achievement, in 2016 it is estimated 36.7 million individuals are now living with HIV and approximately 800,000–1.2 million people died due to AIDS-related deaths. Owing to comparative phylogenetic analysis, the origins of the HIV epidemic are believed to be zoonotic transmission events occurring between select strains of wild chimpanzee simian immunodeficiency virus (SIV) crossing over into human populations [2]. Currently it is understood that this zoonosis might have happened on as many as 4 independent occasions, giving rise to the four classifications of HIV, groups N, O, P, and the pandemic M group [3,4].

HIV-1 is primarily a sexually transmitted virus with transmission occurring through mucosal surfaces. Less frequently, HIV can also be spread vertically by mother-to-child exposure and by direct intravenous inoculation. HIV infection results in the progressive depletion of CD4 T cells, the very cells that orchestrate the critically protective adaptive immune responses to pathogenic infections, until such time as the immune constitution is severely eroded and opportunistic infections ensue. While CD4 T cells serve as the primary targets for HIV infection and replication, not all CD4 T cells are equally depleted. For instance, activated CD4 T cells are more susceptible to productive infection than their naïve counterparts [5]. Initially it was assumed HIV-mediated CD4 T cell depletion was occurring directly via viral cytopathic effects [6]; however, other studies suggested the cells dying in lymph nodes in response to infection were uninfected bystander cells [7]. More recently, a form of cell suicide mediated by caspase-1 dependent pyroptosis was attributed to the massive decline in CD4 T cells [8]. Pyroptosis occurs in non-permissive CD4 T cells (~95% of cells), where infection leads to the accumulation of incomplete reverse transcripts that are detected by endogenous DNA sensor IFI16, leading to inflammasome assembly [8], with the end result being caspase-1 activation and the death of abortively infected cells. This contrasts with permissive CD4 T cells (~5% of cells), in which infection causes the productively infected cell to undergo caspase-3 mediated apoptosis [8]. While pyroptosis is readily detected within infected lymph nodes, it is not seen in peripheral blood T cells, probably due to their lower activation status compared to lymph node CD4 T cells [9]. In fact, the frequency at which and the extent to which HIV viral replication occurs in lymph nodes is between ~5–10-fold higher than in peripheral blood [10]. 

During the very early events post exposure, the HIV viral quasi-species experiences multiple genetic bottlenecks before going on to establish a systemic infection. This dramatic contraction in viral diversity between the sequences identifiable in the donor’s genital secretions, the vaginal mucosa of the recipient, and the systemic compartment is due to a combination of physical and immunological constraints on the virus. The result is a single viral variant establishing systemic infection in >75% of individuals, with multiple variants (<5) involved in >20% of infections [11,12]. Evidence from non-human primate (NHP) studies using atraumatic inoculation of high doses of SIV has provided a blue print for the critical early events during transmission. From such studies, we now know the virus can cross the mucosal epithelium within hours and establish a founder population of infected cells [13]. These founder populations rapidly expand and evolve into larger foci of infection over the next few days, and within the first week the virus spreads to the local draining lymph nodes and becomes a self-propagating infection. During vaginal transmission, vaginal epithelial Langerhans cells (LCs) and dendritic cells (DCs) were identified as the major viral targets for initial infection [13,14,15], while contradicting studies suggested T cells were the key targets for early infection [16,17]. Due to their anatomical locations within mucosal tissues, CD1a^+^LCs and DCs are perfectly situated to encounter invading pathogens quickly [18]. While the migratory properties of antigen-loaded LCs are still being evaluated and appear to depend on the presence/absence of stromal cells and TGF-β secretion [18,19], certain stromal DC subsets encountering HIV can capture and transport virus to the local draining lymph nodes. The main DC population resident within the female reproductive tract (FRT), the CD11c^hi^CD11b^+^CD14^+^ cells co-expressing CD1c, is suggested as important for HIV capture and transport to local draining lymph nodes [20]. 

Through the use of the NHP SIV infection model and phenotyping of infected cells, it has become clear that the early cellular targets for infection are CCR6^+^ CD4^+^ T cells, which expressed the RORγT transcriptional regulator [21], demonstrating that the initial targets for infection were mucosal Th17 cells [21]. Interestingly, although the vagina had the greatest number of virally infected cells, multiple foci of infections were established throughout the reproductive tract, challenging earlier assumptions that the transformation zone and endocervix is the site for viral transmission and infection [17,21,22]. Thereafter, infection spreads systemically via the thoracic duct to various organs of the body including other secondary lymphoid tissues, brain, liver, lungs, and gut [23]. Studies on antiretroviral therapy (ART)-treated NHPs infected intrarectally with SIVmac251 have shown that the latent, replication-competent proviral reservoir is established within the first 3 days of infection (i.e., the eclipse phase) before virus is detectable. This is substantially earlier than previously thought and suggests that a viable preventative vaccine has a very narrow window of opportunity within which to either prevent or contain infection before a pool of latent virus is established [24]. 

## 2. Immune Response to HIV Infection

Once HIV establishes infection, a strong host-mediated immune response is mounted. This initial response is ultimately unable to contain viral replication. As mentioned previously, in the majority of cases, only a single transmitted/founder (T/F) virus is responsible for disseminating infection. In these early stages of infection, the virus effectively enters an evolutionary arms race with the counteracting immune response, driving viral diversity and immune escape. Shortly after infection (~10–15 days), the virus reaches a peak viral load before declining to a viral set point (~30 days), a prognostic marker in the AIDS timeline. This decline in peak viremia coincides with a rapid amplification in anti-viral CD8 T cells, which places a downward selective pressure on the virus [25]. Despite the cytotoxic T lymphocyte (CTL) pressure, HIV evades CD8 T cell control of viremia by rapidly evolving susceptible T cell target epitopes via mutations that affect epitope-HLA binding, mutations that interfere with intracellular epitope processing or mutations that prevent T cell receptor (TCR)-epitope-HLA recognition. This viral evolution acts in concert with Nef-mediated downregulation of polymorphic HLA-A, B, and C molecules on infected CD4 T cells, thereby reducing recognition of infected target cells. Within 6–7 days post-infection, a burst in B cell plasmablast (low affinity short-lived extrafollicular plasma cells with minimal Ig-variable region diversification) numbers occurs, comprising as much as 13% of the circulating B cells in the blood, of which less than only 1.5% has been reported to be HIV-specific [26]. This contrasts with other viral infections like dengue and RSV, in which plasmablast numbers can constitute as much as 30% of the peripheral lymphocyte population, with the majority being virus-specific, strongly indicating that significant indirect B cell activation occurs during early HIV infection [26]. 

Production of plasmablasts signals the introduction of HIV-1 virions or viral antigens into secondary lymphoid structures. Lymph nodes are characterized by the presence of follicles containing IgM^+^IgD^+^ B cells that are separated by an interfollicular region. The T cell zone, containing an abundance of T cells, strategically borders the B cell follicle. During adaptive immune responses, germinal centers form within the B cell follicle, which contains a follicular dendritic cell network (FDC) at its centre [27]. Early after infection, infectious viral particles can be located on the surface of FDCs in the form of long-lived immune complexes. Although FDCs are not known to be productively infected, they are believed to present infectious virus to CD4 T cells that is in close proximity. T follicular helper cells (Tfh) also reside within lymph nodes, a subset of CD4 T cells that is tactically positioned to provide the necessary help to resident B cells and a cell type regarded as a major HIV reservoir. Generally speaking, exogenous antigenic interactions with the B cell receptor (BCR) on the B cell surface result in BCR cross linking, antigen internalization, processing, and presentation in the context of MHC class II. This results in the activation of the naive B cell, which then migrates to the T and B cell zone border for interactions with T cells and to become fully activated [27]. The Tfh cells provide the necessary cognate interactions (CD40-CD40L, MHCII-peptide-TCR) and secreted soluble mediators (e.g., IL-4 + IL-21 cytokines) at the interface between the T and B cell zones [28], the consequences of which are B cell clonal expansion, differentiation, and Ig class switching. Additional co-stimulatory interaction through ICOS and activation of SLAM is important and gives rise to either low affinity plasmablasts, which mature into memory B cells, or it continues to proliferate within the follicle and generate germinal centres [28]. Tfh cells are critically important for the generation of high-affinity GC B cells and to promote the back and forth interzonal migration of B cells for repeated rounds of somatic hypermutation (SHM) [27]. Interestingly, Tfh cell accumulation in germinal centers of secondary lymphoid tissues has been correlated with hypergammaglobulinemia and activated germinal center B cells, with the frequency of Tfh cells secreting IL-21 and IL-4 correlating with the development of broadly neutralizing antibody (bnAb) production [29,30]. 

The early antibody responses do not neutralize the infecting virus, are initially polyreactive, and are directed towards the gp41 glycoprotein before anti-gp120 antibodies are raised [31,32]. Interestingly, priming of anti-gp41 B cell response has been shown to occur in the intestine and in response to intestinal microbiome exposure [33]. This microbiome-gp41 cross-reactive priming may be behind the early gp41 dominated antibody response in early HIV infection [33]. Mucosal antibodies are often referred to as the front lines of immunological defense against pathogens exploiting mucosal surfaces as portals of entry to the body. For this reason, their elicitation through infection or vaccination can be important to ameliorate disease or prevent infection. Within mucosal fluids, IgG, IgM, and IgA antibodies are found, with the latter two isotypes existing as both monomeric and polymeric forms. While the protective properties of mucosal IgA in the context of HIV-1 infection or acquisition prevention have not been proven, elicitation of anti-viral IgA against a number of disease-causing organisms has been shown to augment or correlate with protection. This includes vaccinations against influenza, polio, and rotavirus [34]. Certainly, tantalizing evidence from highly exposed, persistently seronegative individuals (HEPS) suggests that HIV-specific IgA responses correlated with resistance to HIV acquisition [35]. As such, it is widely anticipated that local anti-HIV IgA responses could play a significant role in protection against HIV infection, if present in mucosal secretions prior to transmission. As discussed previously, the early antibody response is directed towards the gp41 portion of Env with the plasma anti-gp41 IgA response being detectable (~13.5 days) before any anti-Gag IgA antibodies (~25.5 days) [34]. Interestingly, during this acute stage of infection, the anti-gp41 IgA response in plasma can be detected during Fiebig stage I/II in 25% of individuals, which then climbs to 50% of individuals by Fiebig stage III. This contrasts with the delayed appearance of anti-gp41 IgA in mucosal samples, which is normally only detectable in ~33% of individuals in Fiebig stage IV [34]. Why such a delay in the appearance of IgA occurs in mucosal secretions is currently unknown. In addition to a delay in the appearance of IgA in mucosal secretions, anti-gp41 IgA antibodies in mucosal secretions have been shown to have a relatively short half-life (~2.7 days) when compared to plasma anti-gp41 IgA (~48.2 days) [34], raising questions as to how to extend the residency of IgA in mucosal secretions when elicited through vaccination. Finally, the early g41-specific IgG response can be found at much higher concentrations (11-fold higher) in genital secretions compared to gp41-speific IgA.

**Figure 1:**
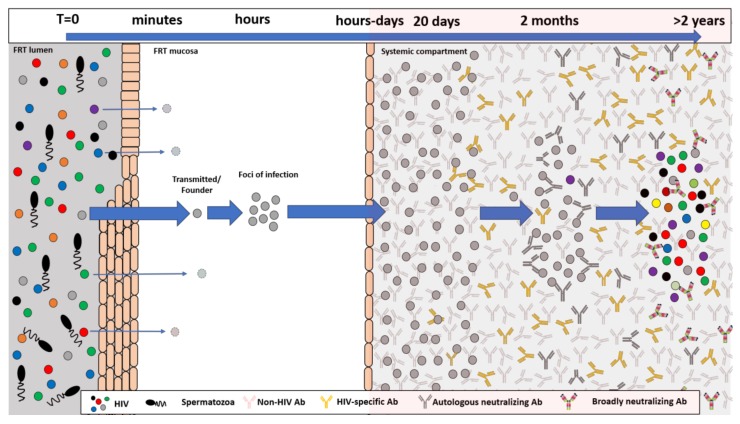
The HIV-1 mucosal transmission bottle neck and developing antibody response. Early after exposure, transmitted founder (T/F) viruses cross from the external mucosal lumen into the mucosal stroma and establish a foci of infection. Within a period of hours to days, the virus then migrates to local draining lymph nodes as either free virus or is carried there by migrating dendritic cells. Within the lymph node, there is an abundance of CD4 target cells to propagate infection, resulting in exponential viral amplification and systemic spread. During these initial few days and weeks, the infecting virus is clonal in nature with little genetic diversification. The early humoral immune response is characterized by the initial development of anti-gp41 antibodies before anti-gp120 are detectable. Over the next few weeks and months, the virus enters into an evolutionary arms race with the developing B cell response, resulting in genetic diversification of the transmitted founder into a viral quasi-species. These viral escape mutants help drive the anti-HIV B cell response and ultimately give rise to autologous neutralizing antibody responses and then to broadly neutralizing antibody responses within a subset of these infected individuals.

Although HIV infection is associated with B cell dysfunction and a blunted antibody response, during early infection autologous strain-specific neutralizing antibodies to the T/F virus and its evolving variants arise within 3–12 months post infection (Figure 1) [36,37]. The generation of B cell lineages producing autologous neutralizing antibody responses forces viral evolution to escape the antibody mediated immune pressure. Ultimately, after a number of years of this continued viral evolution and diversification in response to the relentlessly pursuing B cell immune response, development of bnAbs in ~20–50% of HIV+ individuals can occur [38,39,40,41]. Within individuals generating bnAbs, more than 1 lineage of B cell can be involved, widening the potential of neutralization breadth of the antibody response [42,43]. Despite the generation of circulating bnAbs in certain individuals, their presence is not associated with control of viremia in vivo. However, passive infusion studies in NHPs have clearly demonstrated a protective role for bnAbs against challenge infections [44,45,46,47,48,49]. Why all HIV infected individuals make autologous neutralizing antibodies against the infecting viruses, but less than 50% of HIV+ individuals go on to make any level of bnAb response, is unknown. One reason behind the failure of vaccines to recapture what is seen in natural HIV infection is that host control mechanisms may disfavor bnAb production due to potential antigenic mimicry and autoimmunity. Certainly, many bnAbs have been shown to bind to human proteins or have been identified by diagnostic assays used for determination of autoimmune disease [50,51,52,53]. In addition to autoreactivity, it is well known that bnAbs have extensive levels of SHM and/or long heavy chain complementarity determining region 3 (HCDR3) loops, characteristics of B cells that would normally be eliminated by central and peripheral tolerance [54,55]. What is clear is that bnAb lineages display extraordinary levels of mutations. Under normal circumstances, somatic hypermutation (SHM) and B cell selection enable rapid, high affinity antibody production in a matter of weeks [56]. This involves a small number of variable region mutations in the developing antibody, which can diverge by as much as 5% from the germline sequences. This is in stark contrast to certain bnAbs, in which in excess of 30% of variable region nucleotides can be exchanged [56]. Analysis of VRC01 lineage evolution over a 15-year period identified a rapid rate of bnAb evolution to an early autologous gp120 molecule, which slowed down over time. Despite this deceleration in the mutation rate, antibody evolution appears to continue in response to chronic persistence of antigen, thereby enabling continued SHM [56]. How such extended levels of antigen persistence and B cell evolution can be achieved in the context of prophylactic HIV-1 vaccines remains to be seen.

## 3. Previous HIV-1 Vaccine Efficacy Trials

Since 1987, hundreds of vaccine candidates have been clinically tested as HIV-1 vaccines. However, to date only six HIV-1 vaccine efficacy trials have been completed (Table 1). Most vaccines work through elicitation of protective antibody responses. Furthermore, in a number of disease states, vaccine conveyed immunity has been shown to correlate with both induction and the magnitude of the vaccine elicited antibody titre. Therefore, in the 1990s, initial vaccine candidates were based on Env glycoproteins and tested in preclinical NHP studies [57,58,59], as well as in human safety and immunogenicity trials [60,61,62,63,64]. Such studies provided critical evidence that Env-based vaccines could be safely administered and were immunogenic in humans and NHPs. Yet, studies in NHPs soon identified a significant flaw in early recombinant Env vaccines. Although the elicited immune responses were protective against homologous challenge infections, they were not protective against heterologous challenge [65,66]. In 1999, the randomized, double blind, placebo-controlled efficacy trial of AIDSVAX B/E (VAX003) was initiated and involved the enrollment of 2546 injection drug user (IDU) cohort in Thailand. The AIDSVAX B/E vaccine contained two recombinant gp120 HIV Env antigens from a CXCR4 lab-adapted clade B strain and a CCR5 primary subtype CRF01_AE isolate adjuvanted in alum [67]. Despite induction of anti-gp120 antibodies, VAX003 did not provide any protection from infection, with 8.3% in the placebo and 8.4% in the vaccine arm becoming infected [67]. In VAX003, vaccine efficacy was estimated at 0.1%. Another Env-based efficacy trial named VAX004 was a randomized, double blind, test of AIDSVAX B/B. This formulation was the first phase 3, placebo-controlled efficacy study against HIV acquisition and contained subtype B recombinant gp120 in alum. VAX004 was administered to 5403 men who have sex with men (MSM) and women at high risk of infection in North America and the Netherlands [68]. Despite inducing neutralizing and CD4 blocking antibody in all vaccines, HIV seroconversion rates were 6.7% in the vaccine arm and 7% in the placebo arm, with overall vaccine efficacy estimated at 6% [68]. In short, despite being immunogenic, VAX003 and VAX004 recombinant Env-based vaccines failed to demonstrate any level of protection from infection. 

In contrast to the previous disappointing Env-based efficacy trials, the STEP (HIV Vaccine Trials Network 502, HVTN502) and Phambili (HVTN503) trials were designed to elicit cellular immune responses. This was done as a considerable body of research was being generated; suggested cell mediated immunity was important in the control of both HIV replication and disease progression in long term non-progressors [69,70,71]. This body of evidence was also supported from NHP challenge models [72,73,74]. The STEP study was a phase II, double-blind, randomized, placebo-controlled trial using the MRKAd5 HIV-1 Gag/Pol/Nef vaccine in high-risk of infection, HIV-1 seronegative women and MSM [75]. This multicenter trial enrolled 3000 individuals with study sites in North America, the Caribbean, South America, and Australia [75]. The closely related phase II Phambili trial involved MRKAd5 clade B Gag/Pol/Nef administered to 801 of a scheduled 3000 heterosexual men and women in South Africa [76]. Although both vaccines were immunogenic and well tolerated, an exploratory multivariate interim analysis from the STEP trial revealed an alarming increased incidence of HIV-1 acquisition in male vaccinees versus placebo recipients who had adenoviral seropositivity (5.1% vs. 1.4% per year, respectively) or were uncircumcised (5.2% vs. 1.4% per year, respectively). On the basis of this negative news, both trials were stopped. Interestingly, a follow up sieve analysis was performed on HIV-1 genome sequences from 68 newly infected volunteers from the STEP trial to evaluate if the vaccine exerted any selective pressure on breakthrough viruses. Indeed, a genetic imprint was identified on the founder viral strains. The sieve effect was only seen in predicted epitopes and only in proteins that were components of the vaccine, indicating the possibility of T cell pressure post-infection [77]. Therefore, despite the failure to protect and being stopped early, the STEP trial MRKAd5 HIV-1 Gag/Pol/Nef vaccine was the first to place a selective pressure on the infecting virus.

In 2009, the results from a randomized, multicenter, placebo-controlled efficacy trial involving recombinant canarypox vector (ALVAC-HIV) plus two recombinant gp120 boosts (AIDSVAX B/E) were released [78]. The RV144 “Thai Trial” enrolled 16,402 healthy individuals at heterosexual risk of HIV acquisition, who were divided into vaccine and placebo arms. In the intention-to-treat analysis, a trend towards prevention of HIV-1 infection among vaccine was seen (vaccine efficacy = 26.4%). However, in the modified intention-to-treat protocol, in which seven individuals were removed from the study for being HIV+ at initiation of the study, vaccine efficacy rose to 31.2% [78]. Interestingly, a *post hoc* analysis in behavioral risk and vaccine efficacy revealed an early peak in vaccine efficacy estimated to be 60.5% in the first year, which declined to 31.2% thereafter [79]. Protection in this study was attributed to development of non-neutralizing IgG against the V1/V2 region of HIV-1 Env and not the low-level induction of neutralizing antibody. Comparing the genetic sequences of breakthrough viruses in vaccine recipients by sieve analysis identified that HIV strains isolated from vaccinees had genetic signatures of vaccine-induced immune pressure within V1/V2 [80]. The escape depended on class I HLA A*02 and A*11 restricted epitopes in the recombinant gp120, with vaccine efficacy greater in A*02+ (54%) individuals than A*02− (3%) individuals [80]. Within A*02+ individuals, vaccine efficacy of breakthrough viruses containing K169 within V1/V2 was 74% compared to A*02− with 15%, thereby emphasizing that HLA A*02 may have played a significant role in RV144 and the overall importance of HLA genotypes in HIV vaccine trials [80]. Finally, the V2 binding antibody from vaccines was able to mediate ADCC activity, and this was dependent on K169 in the breakthrough Envs [81]. 

More recently, the randomized, double-blind, placebo-controlled efficacy trial (HVTN505) of the Vaccine Research Centre’s (VRC’s) DNA/Ad5 HIV-1 vaccine was halted by recommendations from the data and safety monitoring board for lack of efficacy. The trial enrolled 2504 men and transgender women who have sex with men to receive either vaccine (*n* = 1253) or placebo (*n* = 1251) [82]. In this study, the baseline Ad5 serum neutralizing antibody titre was to be less than 1:18, and men had to be circumcised. The DNA vaccine contained clade B *gag*/*pol*/*nef* and clade A, B, and C *env* while the recombinant Ad5 expressed clade B *gag*-*pol* and clade A, B, and C *env*. HIV acquisition was detected in 27 vaccinees at week 28+, while only 21 infections were recorded in the placebo arm (vaccine efficacy = −25%) [82]. Overall, 41 individuals became HIV+ in the vaccine arm, while 31 became infected in the placebo arm, supporting a lack of vaccine efficacy [82]. Despite an increase in HIV infection in the vaccine arm vs. placebo, the number of infection in the week 28+ primary analysis and the total number of infections in the modified-intention-to-treat analysis revealed no statistical significance [82]. Subsequent analysis of breakthrough viral genomes in vaccinees vs. placebo recipients revealed that HIV-1 diversity was significantly lower in *gag*, *pol*, *vif,* and *env* genes, with Env sequences significantly more distant from the subtype B vaccine insert in vaccines [83]. Interestingly, these signatures of immune pressure were mapped to the CD4bs [83]. Since the sieve analysis was identified primarily in Env, it is argued that the selective pressure on breakthrough virus in vaccinees was in Env regions associated with infectivity, with indications that antibodies might be partially responsible. Taken together, this suggests that although no protection from HIV acquisition was seen (i.e., no vaccine efficacy), the vaccine might have induced a rapid Env diversification post-HIV acquisition due to elicited immune pressure, or the vaccine did protect against HIV acquisition from strains that were more closely related the vaccine strains [83]. 

In summary, of the six HIV-1 vaccine efficacy trials that have been carried out, only the RV144 study has demonstrated a modest reduction in HIV-1 infection rates using a modified intention to treat protocol. Post Hoc analyses on the STEP and HVTN505 trials revealed enhanced viral evolution in response to vaccination, suggesting immune pressure was being exerted on the virus. Collectively, a great wealth of information has been accrued from these studies, with vaccines based on just viral vectors or heterologous viral vector prime protein-boost protocols appearing to perform better than multi-dose protein-based vaccines. A rich HIV-1 vaccine clinical trials pipeline, along with the initiation of HVTN702, a repeat of the RV144 trial in South Africa, provides much hope that additional correlates of protection will be elucidated and that an HIV-1 vaccine might yet become a reality. 

## 4. Preclinical Evaluation of Novel Protein Based Immunogens

Shortly after establishing that the HIV retrovirus was the cause of AIDS, the field was wildly optimistic that a vaccine would be quickly and easily found to either prevent or alter the disease course, a promise that has not yet been fulfilled. After some very early work in the newly established non-human primate models that used various *nef* or multiple gene deleted viral strains, it was clear that the live-attenuated vaccine strategy was unlikely to produce an acceptable vaccine, as the virus demonstrated great powers of deletion repair and enhancement of viral fitness [84,85,86]. Next, vaccine development studies concentrated on creating a subunit vaccine, a strategy that was considered to have major advantages in terms of potential safety profile over the more traditional live-attenuated or even a heat-killed whole virus vaccine. Work focused on producing recombinant proteins of various Env gp160 or gp120 versions from various viral strains and clades, as well as the assessment of the immunogenicity of such proteins produced from a multitude of gene expression (mainly viral) systems. Indeed, pre-clinical studies have examined the potential of countless permutations of vaccination regimens consisting of expression vectors, recombinant proteins, and delivery schedules, as well as delivery methods and routes. These pre-clinical studies fed through to clinical trials, and it has become increasingly clear that while it is relatively straightforward to elicit appreciable immune responses in people, the immunity generated by the first or second generation vaccine proteins is not protective and lacks neutralization breadth. What has also become clearer over the intervening years is that a relatively large percentage (10–30%) of infected people are able to produce bNabs and, indeed, about 1% will go on to naturally produce antibodies that are extremely broad and that are highly potent, and are effective at very low serum levels [87,88]. Therefore, the natural experiment has been performed; people can make potent and broadly neutralizing antibodies, which is particularly impressive, as these are generated in the setting of infection and a potentially waning immune system. These observations have pushed the field of vaccinology to focus on antigen design to produce proteins that most accurately reflect the native HIV Env trimer and also to target the pathway of bNab development from the early reactive germline sequences to the final hypermutated bNab clones. 

The Env protein present on a viral particle can exhibit a number of structural forms, whether due to the conformational movements typical of all protein complexes or the various intermediates caused by co-receptor binding and the fusion of the viral Env to the cellular receptor, or from the overall metastability of the trimeric protein complex. In addition, the viral particle ages the surface glycoproteins, which undergo degradation by proteases, in which even a small break in the protein chains can lead to a dramatic loosening of the integrity of the trimer, in which torsional forces in the Env structure distort the molecule presenting a large (currently undefined) number of Env structure variants to the humoral immune system [89].

**Figure 2:**
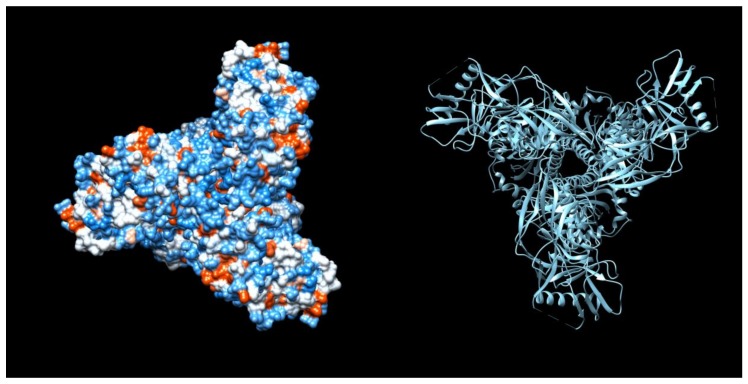
Structural models of the BG505.SOSIP.664 HIV Env protein. The HIV-1 BG505.SOSIP.664 protein PDB data file (PDB file 4ZMJ: Crystal Structure of Ligand-Free BG505 SOSIP.664 HIV-1 Env Trimer [91,92]) was imported to UCSF Chimera program [93] to visualize the molecular structure. The hydrophobicity model on the left (looking at the protein from the top) shows the typical propeller-shape of the BG505.SOSIP.664 HIV Env trimeric molecule, while the ribbon diagram on the right gives detail of the positions of the beta-pleated sheets, alpha-helices and loops that make the structure. The BG505.SOSIP.664 HIV Env exhibits a mature and pre-fusion closed trimer, a conformation recognised by bnAbs that would be expected to target the native trimer presented on infectious HIV. Further design modifications of the BG505 molecule and other Envs have increasingly succeeded in creating stabilised and more native-like trimer structures that are likely to be the next generation vaccine candidates to generate bnAbs.

This heterogeneity of potential Env immunogens pushed the field to accelerate the design and development of stable HIV-1 Env trimers that are soluble and have a native trimer tertiary and quaternary structure. These designer Envs incorporated a number of stabilizing mutations and cross-linkages that forced the protein into a native-like shape, mostly retaining the glycan moieties but essentially locking the Env into a stable non-infectious trimer (Figure 2). The primary exemplar of the optimal native-like trimer to date is the BG505 SOSIP.664 molecule, although this recombinant molecule has recently been shown to possess an unusual ‘glycan hole’ shared by only 3% of global isolates, allowing access of bnAbs to the native-like structure [90]. These glycan holes are present in many HIV Env isolates, at various glycosylation sites, and are potential weaknesses to target in the Env glycan shield. However, while it is apparent that many designed Env trimers, as well as Env isolates, have these alterations in the glycosylation residues to create holes in the ‘glycan shield’ that allow the generation of neutralising tier 2-type antibodies, it has been observed that these Env generate sera with reasonable autologous potency but little heterologous or breadth of activity. Therefore, while these designed Env are clearly highly native and demonstrate strong structural integrity, they have not yet been able to elicit bnAbs of the type that can be isolated form infected patients.

Of the six well described broadly neutralising Ab clusters in the trimer HIV Env molecule, three are highly dependent on the quaternary structure of the Env and also have a strong bias for binding to both protein and glycan elements of the Env structure. Depending upon the final trimer quaternary conformation, there can be more or less exposure of glycan moieties leading to higher or lower degrees of glycan processing, and therefore the final protein-glycan complex can appear strikingly different to a potentially reactive B cell from just a small relaxation or tightening of the overall trimer. Indeed, some 50% of the mass of the HIV Env trimer are glycans, most of which are of the incompletely processed high-mannose type, indicating that the constrained structure of the trimer complex is protecting these glycans from being processed into complex sugars during golgi transit.

Rational vaccine antigen design has been informed by the isolation of a number of bnAbs against the HIV Env protein; these naturally occurring antibodies are effectively telling us what we need to make, and, of course, each one of these antibodies contains a ‘negative imprint’ of a part of the Env molecule and the tertiary structure of that epitope. It is clear that the continued isolation and analysis of these bnAbs will further advance our knowledge of the antigen–glycan structures required that match the final matured and somatically hypermutated bnAb clone, but these observations will not necessarily inform us as to how the bnAb clone developed. The heavy and light chain variable domain (VH and VL) family usage of a bnAb will tell us the preferential antibody (Ab) gene family usage of the germline ancestor. However, for HIV it has been shown that most germline ancestors do not in fact bind to HIV Env [94] and have undergone a high degree of somatic mutations, insertions, or deletions, though logically the germline progenitor must have bound to one of the versions of HIV Env to have been initially selected [38,95,96,97]. Furthermore, a vaccine antigen that may effectively stimulate a bnAb germline precursor may be quite different from an Env carefully designed to replicate the native-like and infectious trimer, and thus an effective vaccine regimen may require multiple designed immunogens (native-like or not) to drive bnAb development and maturation [98]. Recent work utilizing germline reverted bnAbs to screen mammalian cell expressed libraries of BG505 related gp140/gp120 molecules has identified a number of stabilized HIV Env trimers that bind to both germline and mature bnAb versions [99]. Furthermore, several key studies have detailed that structural and/or glycan heterogeneity, localized precursor frequency, and BCR affinity each have an interdependent role to play in the stimulation and expansion of bnAb precursors [100,101]. This work is highly likely to lead to a clearer definition of the key protein/glycan moieties that trigger the initial antigen-specific B cell activation that leads to the fully mature bnAb capable of neutralizing the virus, and it will be critical to move these designed Env, capable of stimulating precursor bnAbs and promoting their continued maturation into high affinity viral neutralisers, as quickly as possible into clinical trials. 

## 5. Inferring from Transmitted Founder (T/F) Viruses to Guide HIV Vaccine Development

One of the major goals for HIV vaccine development is to prevent HIV-1 acquisition at mucosal surfaces. Therefore, understanding the mucosal infection process is critical and is receiving much attention. During sexual transmission of HIV-1, the donor exposes the recipient to a viral swarm consisting of billions of copies of genetically diverse virus. In the vast majority of cases (60–80% of infections), a single virus successfully evades mucosal secretions in the mucosal vault, crosses the mucosal epithelium, binds to and infects a susceptible target cell, avoids immune mediated clearance, and goes on to establish a systemic infection. The fact that a single virus successfully overcomes these host-obstacles out of the billions of virions the recipient is exposed to suggests this is a relatively inefficient process. As such, questions surrounding the success of transmitted/founder (T/F) viruses are being asked. Is this just a stochastic event where every virus has an equal opportunity to establish infection, or are there phenotypic properties associated with T/Fs that predispose them towards successful infection? It is important to understand that nature has provided us with the necessary proof of concept that bnAbs responses can be generated in humans and that autologous neutralizing antibody responses are precursors to the bnAb response. Therefore, the T/F Envs and related breakthrough viruses may provide essential insights into novel vaccine strategies and improved immunogen design.

In a study involving heterosexual transmission pairs from largely monogamous cohabitating couples in Zambia, Derdeyn et al. evaluated the nature of heterosexually transmitted HIV-1 infection in a bid to better understand the infecting virus. In seven transmission pairs, the transmitted virus clustered with subtype C reference strains, and in one transmission pair, infecting virus clustered with subtype G [102]. They evaluated a 257 nucleotide sequence stretch spanning positions 391–1254 of the HIV Env (HXB2 numbering) coding region and then focused only on sequences that spanned the V1–V4 region. By comparing the frequency of sequences below at or above the median donor length, recipients from 6 of the 8 pairs had V1–V4 lengths that were below the donor median [102], suggesting that viruses encoding Env glycoproteins with shorter V1–V4 regions may be transmitted more easily or are fitter in recipients. Further analysis revealed the transmitted virus in 5 of the 8 pairs contained a statistically significant lower number of potential N-linked glycan (PNLG) sites than the median number in the corresponding donor [102], thereby providing tantalizing evidence that T/F viruses may indeed express certain phenotypic characteristics that distinguishes them from the rest of the inoculating viral swarm. A subsequent study in Kenya evaluated the viral Env V1–V2 length and looked for evidence of reduced N-linked glycosylation in viruses samples from 27 women and eight men within 70 days of heterosexually acquired infection [103]. Again, the subtype A sequences from early infection were found to have significantly shorter V1–V2 loop sequences and fewer potential N-linked glycosylation (PNLG) sites [103], collectively strengthening support for shorter hypervariable regions, with fewer PNLG sites being important for T/F viruses. Conversely, in the later study, the T/F viruses from a cohort of 13 men and women infected with subtype B HIV-1, within 142 days post seroconversion, did not show evidence of having shorter V1–V2 sequences than those found in the Los Alamos database. Furthermore, they did not find any reduction in the number of potential PNLG sites, indicating that shorter hypervariable regions and fewer N-linked glycan might not play a role in mediating HIV transmission in subtype B infection [103].

Most HIV-infected patients develop neutralizing antibody responses (nAb) against the autologous infecting strain early in infection. However, this nAb response displays no cross-strain neutralizing activity. To better understand how T/F viruses can generate neutralizing antibody responses, Li et al. evaluated the course and magnitude of the nAb response against Env glycoproteins present at acute and early infection with subtypes B and C HIV-1 [104]. While the course of nAb responses in plasma were similar between the two cohorts, subtype C infected individuals developed plasma nAb responses that were 3.5-fold higher than those seen in subtype B-infected individuals [104]. Critically, the higher titres in the subtype C cohort were associated with viruses having significantly shorter V1–V4 amino acid lengths with fewer glycosylation sites than the B cohort [104]. The fact that subtype C virus triggered autologous nAbs and that the T/F virus had shorter and less glycosylated Envs suggests this phenotype could be exploited for rationale vaccine design. However, for an efficacious clade C vaccine to be realized, increasing the neutralizing breadth of the nAb response would be necessary.

The HIV Env spike is a metastable complex composed of surface gp120 subunits noncovalently linked to gp41 transmembrane domains. The trimerization of the Env complex are the result of noncovalent interaction between the gp41 subunits with additional interactions arising from gp120-gp120 contacts near the trimers apex [105]. The Env oligomers function is to facilitate target cell binding and entry by engaging CD4 and the coreceptor CCR5. As with other viruses, surface Env density plays an important role in mediating cell fusion. However, HIV is set apart from most viruses in that there is a relative sparsity of intact Env trimers displayed on the viral surface (~7–14 spikes/virion), and spontaneous shedding of Env oligomers is known to occur. As such, HIV displays far fewer Env than other viruses such as SIV (~70 spikes/virion), Influenza (~300 spikes/virion), Adenovirus (240 hexon trimers/virion), and Hepatitis B Virus (~120 spikes/virion) [106]. The precise numbers of intact surface Envs necessary to mediate target cell infection is not known, but various reports range from 1–8 trimers [107,108,109,110]. Therefore, it is important to note that T/F viruses have been described to display a greater Env density than viruses analyzed at later timepoints in infection. A study by Gnanakaran et al. searched for amino acid signatures in subtype B Env sequences that were associated with transmission and sequences that were recurring in chronic infection [111]. By assessing thousands of sequences from hundreds of patients, a number of signatures were identified as promising. The first was located at position 12 in the Env signal peptide (SP), and the second was the loss of an N-linked glycosylation site at position 413–415 [111]. Both signatures were described to potentially influence Env expression and incorporation into virions. The Env SP is fairly long (~30 amino acids) and contains a polar N-terminus followed by a hydrophobic core. The role of the SP is directing Env in its translocation to the endoplasmic reticulum (ER), where it undergoes, folding, glycosylation, and trimerization. When compared to the cleavage of the SP of other glycoproteins, the cleavage of the HIV SP is slow [112,113]. Therefore, a histadine (H) at position 12, despite being present in both early and chronic viruses, was found to be statistically significantly enriched in early viruses compared to chronic viruses, which in the latter case was commonly mutated to arginine (R) or proline (P). As for loss of a probably N-linked glycosylation site (PNLG) at position 413–415, a mutation away from 415Threonine (T) was found to be enriched within acute samples or early after infection. The 415T is part of the PNLG sequon at N413 and is normally glycosylated [111]. This is near the c-terminal end of the V4 loop and proximal to the CCR5 coreceptor binding site and CD4 binding site. Critically, PNLG at 413–415 correlates with reduced b12 binding [111]. This work was supported by a computational approach described by Asmal et al., who showed that in T/F viruses, 415H or a similar positive charged amino acid such as arginine (R), as opposed to non-basic residues at this locus, is associated with higher Env expression and incorporation into virions [114], thereby further setting the stage for phenotypic differences in T/F viruses compared to chronic viruses. Interestingly, Parrish et al. found that clade B and C T/F viruses contained 1.9 times more Env per unit of reverse transcriptase (RT) activity than chronic viruses, and that T/F viruses were 1.7-fold more efficiently captured by monocyte derived DC [115]. It could be speculated that higher Env density on T/F viruses may help them to bind and infect both tissue resident and infiltrating CD4 T cells, thereby giving them a higher chance of establishing infection. It is also plausible to assume that HIV modulates its glycan content to evade immune responses and to also specifically target mucosal resident DCs for lectin-mediated binding (e.g., DC-SIGN) and transport them to regional draining lymph nodes. Taken together, although still a controversial topic, T/F viruses appear to have significant phenotypic properties that could be exploited for the purposes of an efficacious HIV-1 vaccine. From an immunological and vaccine point of view, understanding Env density and the glycan shield on the HIV virus is critical, as it influences B cell activation through BCR cross linking and down-stream processes such as clonal expansion and antibody affinity maturation, thereby giving potential insights into how autologous neutralizing antibodies might be generated and what is special about the individuals that generate broadly neutralizing antibody responses. In essence, studying the continual arms race between infecting virus and the humoral immune response could illuminate antibody lineages and their associated viral escape mutants that eventually allow specific Envs, amongst the circulating quasi-species, to promote autologous and broadly neutralizing antibody production. 

With that in mind, McCurly et al., constructed and tested T/F based vaccine constructs using sequential clade C Envs from a south African Individual (CH0505) known to elicit CD4bs neutralizing antibody in NHPs [116]. The CH0505 T/F Env, week 53, week 78, and week 100 Envs were presented by virus like particles (VLPs) with or without co-administered gp120. It was speculated that the conformation of the T/F Env would enable the correct angle of approach for engagement of B cells targeting the CD4bs [116]. The outcome was neutralization of autologous tier 2 T/F virus, but the vaccine had no breadth for other tier 2 viruses despite Abs being mapped to the CD4bs. This study demonstrated that using sequential Env vaccinations could initiate a B cell lineage with potential to evolve towards heterologous neutralization [116]. 

By studying viral-host coevolution over a 0–5 year period of a clade C infection, Bonsignori et al. were able to identify key events in the ontogeny of V3 glycan bnAb response within a Malawian individual CH848. Sequential sampling of the virus over this time revealed neutralization was restricted to virus that contained an N332 N-linked glycosylation site [117]. In this instance, the length of the V1 loop of the T/F virus was 34 residues, while the average V1 amino acid length in the Los Alamos database was 28 residues. The viral quasi-species was then shown to undergo a transition from a long V1 loop in the T/F to a short loop (~16–17 amino acids) when escaping from the autologous neutralizing antibody response [117]. This truncated V1 loop caused the expansion of the DH270 bnAb lineage, placing significant pressure on the virus, causing it to select for viral escape mutants with longer V1 loops. This ultimately increased bnAb breadth by enabling DH270 to recognize a broader spectrum of Envs. It is noteworthy that the DH270 unmutated common ancestor did not bind the T/F Env but did bind to peptides from the base of the V3 loop [117]. Thus, vaccine priming with T/F virus Env, V3 based structures, or peptide antigens from V3, followed by Envs with progressive longer V1 lengths, might induce V3-glycan bnAbs [117]. Indeed, changes to the V1/V2 length over the course of HIV infection have previously been reported to increase along with the number of PNLG sites [118]. This latter example highlights that sequential vaccinations strategies with multiple Env modalities might be necessary to harness the necessary B cell lineages that can lead to the generation of bnAbs. 

In another study, vaccination of NHPs with a cocktail of gp140 oligomeric Envs from an HIV-1 T/F and 6 of its variants, all derived from an HIV-1 infected African (CAP206) who generated bnAbs against the gp41 MPER region, was carried out [119]. In this study, potent neutralizing antibody titres were generated against tier 1 viruses in all animals, while the vaccine also managed to produce a neutralizing antibody lineage that had tier 2 neutralization in some animals [119]. The neutralization profile resembled the plasma response seen at 6 months within the CAP206 individual but was shown to bind to the V5 region of Env, thereby demonstrating that T/F Env and variants can generate autologous tier 2 neutralizing antibody [119]. 

Collectively, these observations indicate that the phenotypic properties of T/F viruses appear sufficiently distinct from the rest of the viral swarm and that their Env glycoproteins could be important for a protective vaccine. Future vaccine studies investigating sequential vaccination strategies, starting with T/F Envs and finishing with accurately constructed Env trimers, might induce the necessary B cell ontogenesis by immuno-focusing on the necessary structural components that culminate in bnAb responses.

## 6. Ongoing Clinical and Preclinical Testing of HIV Vaccines

Viral vectors are the best delivery tools for vaccine development, because they have intrinsic adjuvant capability and unique cellular tropism, and they are able to trigger robust adaptive immune responses. A number of new vectors, including Ad26 (adenovirus serotype 26), Ad35, poxvirus (e.g., canarypox virus-based ALVAC vector), replication competent Ad4, and CMV vectors, are currently being developed (Table 2) [120,121,122,123,124]. However, viral vector vaccine platforms do have some limitations, such as preexisting immunity. It is also noticed that different vectors, even derived from phylogenetically similar viruses, can elicit substantially distinct immune responses, in terms of quality, quantity, and location, which can ultimately affect protection efficacy. The two-viral vector-based vaccine trials, Imbokodo and HVTN 702, have resulted from years of scientific testing and clinical development, currently representing the best efforts in HIV vaccine development. Both HVTN 702 and Imbokodo trials are described in more detail below.

### 6.1. HVTN702

The HVTN 702 study is a Phase 2b/3 clinical trial based on the modestly protective RV144 clinical trial [78,125]. The vaccine regimen consists of two experimental vaccines: a canarypox-vector based vaccine called ALVAC-HIV and a two-component gp120 protein subunit. Based on the vaccines used in RV144, both ALVAC-HIV and the protein subunit have been modified to be HIV subtype C-specific. In addition, MF59 (an adjuvant different from the one used in RV144) was combined with the protein component in order to elicit a more robust immune response. Finally, the participants will receive booster shots at the one-year mark in the hope of prolonging the early protective effect observed in RV144. 

The HVTN 702 study is the most advanced and largest HIV vaccine clinical trial initiated thus far and will be carried out in South Africa. It will enroll 5400 healthy, sexually active men and women aged 18 to 35 years old. Half of the participants will be randomly assigned to receive the experimental HIV vaccine regimen, and the other half will receive a placebo. A total of five injections will be given to participants over one year and then will be followed up for another two years. Results from the study are expected in late 2020.

### 6.2. Imbokodo Efficacy Trials

Imbokodo is a Phase 2b proof-of-concept study, and the vaccine regimen is based on “mosaic” immunogens in an effort to induce immune responses targeting the diverse global HIV strains, which differs from HVTN 702 study. Prior studies in NHP with these mosaic-based vaccines were able to protect monkeys from SHIV challenge [126]. Furthermore, two early-stage human clinical trials, APPROACH and TRAVERSE, showed that these vaccines are well-tolerated and can generate HIV specific immune responses in the vaccinees. Based on the findings from these two early-stage clinical trials, a lead candidate regimen was selected for further evaluation [127]. 

The APPROACH study was a phase I/II clinical trial initiated in Dec. 2014 and was scheduled for completion in April 2019 [128]. It enrolled 400 participants (18–50 years). The purpose of this study is to evaluate the safety/tolerability of different regimens containing Ad26.Mos.HIV, Modified Vaccinia Ankara (MVA)-Mosaic, and/or HIV-1 Clade C gp140 drug product (gp140 DP) components through intramuscular route and to compare Env specific antibody responses between the different vaccine regimens. 

The TRAVERSE study is a double-blind clinical trial phase I/II started in Jun. 2016 and completed in May 2018 [129]. It enrolled 198 participants (18–50 years) in the United States and Rwanda. The purpose of this study is to assess the safety/tolerability of the 2 different vaccine regimens; it first aims to prime with trivalent Ad26.Mos.HIV and boost with trivalent Ad26.Mos.HIV and Clade C gp140 plus adjuvant; secondly, it aims to prime with tetravalent Ad26.Mos4.HIV and boost with Ad26.Mos4.HIV and Clade C gp140 plus adjuvant. Interim data indicate that both are well-tolerated and can elicit anti-HIV immune responses [127]. 

The Imbokodo trial is to evaluate the quadrivalent mosaic vaccine based on the TRAVERSE study. It will enroll 2600 HIV-negative women in sub-Saharan Africa. All participants will receive four vaccinations of either the experimental vaccine regimen or placebo over one year. The final two doses will be given together with an HIV clade C gp140 protein and an adjuvant (aluminum phosphate). Participants will be followed up over two years. Results from Imbokodo are expected in 2021.

The ASCENT trial is another ongoing adenovirus vector-based HIV mosaic vaccine trial. It is a double-blind phase I/II clinical trial initiated in Mar. 2017. It enrolled 150 participants (18–50 years). The primary purpose of the study is to assess safety/tolerability and Env-specific antibody responses of two different mosaic-based vaccine regimens, i.e., Ad26.Mos4.HIV vaccine through intramuscular route at Week 0 and 12, followed by Ad26.Mos4.HIV vaccine + Clade C glycoprotein 140 vaccine containing 250 mcg (microgram) of total protein mixed with adjuvant (aluminium phosphate) at Week 24 and 48, or Ad26.Mos4. HIV vaccine at Week 0 and 12 followed by Ad26.Mos4.HIV vaccine and a combination of 125 mcg Mosaic gp140 and 125 mcg Clade C gp140. Results from ASCENT are expected in early 2019 [130].

## 7. Application of Systems Biology and Serology for Improved Vaccines

Systems serology approach offers an unbiased and comprehensive approach to systematically survey humoral immune responses, capturing the array of functions and humoral response characteristics that may be induced following vaccination with high resolution. Coupled to machine learning tools, large datasets that explore the “antibody-ome” can help in the identification of features associated with humoral immunity that distinguish protective from non-protective responses [131]. Systems serology is able to associate antibody features and functions with protection from HIV infection, which is now becoming a powerful tool with which to investigate the humoral immune system. 

A number of different data-driven computational approaches or “machine learning” have been applied to systems biology, systems vaccinology [132,133], and, more recently, systems serology [134]. These applications have identified correlations between certain immune signatures and vaccine efficacy for a number of diseases such as yellow fever [135], influenza [136], and malaria [137,138]. Chung, et al. utilized a variety of modelling and data clustering techniques to outline the antibody profiles of two non-efficacious HIV vaccine trials: VAX003 (AIDSVAX B/E [67]) and HVTN204 (DNA/rAD5 [139]) [140], and the moderately protective RV144 trial (ALVAC and AIDSVAX B/E [78]). Application of correlation networks analysis to these different vaccine trials revealed that, in RV144 trial, Env-specific IgG1 and IgG3 responses were correlated with multiple antibody effector functions, such as ADCC, ADCP, and ADCDC. On the contrary, these correlations were not found in the two non-efficacious vaccine trials. Furthermore, they used the immune correlate analysis to separate RV144 recipients into two groups with either high or low V1/V2 responses [141]. As expected, the former, with predicted low risk of infection, had high HIV-specific IgG1 levels and polyfunctional Fc effector functions including ADCP, ADCC, and antibody-dependent NK cell activation. On the other hand, the low IgG V1/V2 responders, with predicted higher risk of infection, were correlated with HIV-specific IgA, which is consistent with previous immune correlate studies [141]. This includes the analysis of risk of infection by Tomaras et al. that revealed plasma IgA/IgG ratios were higher in infected individuals than in uninfected vaccine recipients and that Env-specific IgA antibodies from RV144 vaccinees could block binding of ADCC-mediating mAb to HIV-1 gp120 [142]. Further analysis showed that the high IgG3-V1/V2 responders were associated with activating FcγRIIa and FcγRIIIa, receptors required for ADCP and critical for NK cell activation and ADCC, respectively, which was also confirmed to be associated with protection by other studies [143]. Although IgA has been correlated with lack of protection in RV144, it is important to note that other studies have identified protective effects of vaccine-elicited anti-HIV IgA [144]. Therefore, it is worth mentioning that a phase I, double blinded study published by Leroux-Roels et al*.* and conducted in 24 healthy HIV-uninfected women demonstrated the safety, tolerability, and immunogenicity of a virosomal vaccine expressing HIV-1 gp41-derived P1 lipidated peptides (MYM-V101) [144]. In this study, vaginal and rectal P1-specific IgG was induced, and in ~50% of the participants vaginal anti-P1 IgA was elicited. Although the induced vaginal IgA was non-neutralizing, it was shown to inhibit HIV-1 transcytosis, thereby providing evidence that anti-HIV IgA responses may be protective against HIV transmission. Taken together, the systems serology and related bioinformatics analyses provided an ideal tool with which to study the network for Fc functions and their pathway analyses [145,146].

Systems serology approaches in non-human primate (NHP) models and efficacy trials: Several SIV and SHIV vaccine trials have also adopted systems serology approaches to analyze the vaccine efficacy [147,148,149]. One example is an NHP study with an RV144-like vaccine consisting of an ALVAC prime-followed by gp120 protein boost vaccine strategy. This study was analyzed by a combination of systems biology and systems serology, which revealed the association of RAS activation, mucosal IL-17-producing innate lymphoid cells, and V2 antibodies with delayed acquisition of infection [149]. In another NHP vaccine study, an Ad26 prime and Env protein boost resulted in 50% protection efficacy from repeated SIV challenges. The systems serology analysis revealed that this protection was associated with polyfunctional Fc effector functions including ADCP, ADCC, and ADCD, as well as antibody-mediated NK cell activation [147]. 

The systems serology is still in its early developmental stages, and there is no doubt that it will be further improved to better characterize comprehensive humoral immune responses. However, by using systems serology, we have already been able to dissect the humoral immune responses in NHP vaccine trials which provide us with a unique opportunity to understand the vaccine-elicited immune responses to a much deeper degree. These approaches will eventually enable us to build parallels between NHP and human protective responses, and help us design better vaccines for human trials.

## 8. Summary

Although over 35 years have passed since the discovery that HIV-1 was the etiological agent behind the AIDS epidemic, a preventative vaccine has not yet been realized. During that time, significant conceptual and technological advances have been made in HIV vaccinology, resulting in efficacy studies of candidate vaccines that aimed to reduce the likelihood of HIV acquisition or to increase the immune pressure on the virus and its escape mutants. Collectively, these studies have provided critical insights into the potential correlates of protection that have been missing for decades. HIV-1 is a highly mutable virus owing, in part, to its rapid replication and low fidelity reverse transcriptase activity. From a historical standpoint, vaccinology has been successful at preventing infections from organisms that express stable/invariant antigenic structures that can be targeted by antibodies. For this reason, the HIV-1 virus has essentially presented vaccine researchers with a “moving target” for which to create a vaccine, necessitating the development of novel vaccine approaches. It is important to understand that, despite the history of failures in HIV vaccine development, the preclinical and early phase clinical vaccine pipeline is rich in both novel and diverse anti-HIV vaccine strategies. This, coupled with the on-going efficacy trials designed to improve upon RV144, leads many to see a bright future for HIV vaccine development. 

## Figures and Tables

**Table 1:** Previous HIV-1 vaccine efficacy trials.

Trial ID	Vaccine Description	Phase	Number of Participants	Year	Results
AIDSVAX B/E (VAX003)	Two clade B and one CRF01_AE gp120 antigens in alum	III	2546	1999.3–2003	No protection
AIDSVAX B/B (VAX004)	Clade B recombinant gp120 antigens in alum	III	5417	1998.6–2003	No protection
HVTN502 (STEP)	MRKAd5 HIV-1 Gag/Pol/Nef	IIb	3000	2004.12–2007.9	Halted at interim analysis for futility; early transient increased infection in vaccinees
HVTN503 (Phambili)	MRKAd5 clade B Gag/Pol/Nef	IIb	801	2007.1–2007.9	No effect, late increased HIV infection in unblinded male vaccinees
RV144	ALVAC-HIV vCP1521, AIDSVAX B/E rgp120 in alum	III	16,402	2003.10–2006.7	31.2% protection
HVTN505	DNA, rAd5 (A, B, C)	IIb	2504	2009.6–2017.8	No protection

The previously completed human efficacy trials designed to prevent HIV acquisition are shown. Ad = Adenovirus; gp = glycoprotein; HVTN = HIV Vaccine Trials Network; MRK = Merck; MVA = modified vaccinia virus Ankara; NCT = National Clinical Trials identifier; vCP = canarypox vector; CRF = circulating recombinant form.

**Table 2:** Recent and ongoing HIV clinical trials.

Trial ID	Vaccine Description	Category	Phase	Duration
NCT01084343	Virosome (IRIV) expressing lipidated gp41 peptide	Virosome based	I	2009.11–2010.09
RV305	ALVAC-HIV (vCP1521) and/or AIDSVAX gp120 B/E late boost	RV144-related	II	2012.04–2017.05
RV306	ALVAC-HIV (vCP1521) prime, ALVAC-HIV/AIDSVAX gp120 B/E boost	RV144-related	II	2013.09–2017.11
RV328	AIDSVAX gp120 B/E prime and boost	RV144-related	II	2014.07–2018.12
HVTN100	ALVAC-HIV (vCP2438) prime, ALVAC-HIV (vCP2438)/bivalent clade C gp120/MF59 boost	RV144-related	I/II	2015.01–2017.01
HVTN702	ALVAC-HIV (vCP2438) prime, ALVAC-HIV (vCP2438)/bivalent clade C gp120/MF59 boost	RV144-related	IIb/III	2016.10–2021.07
X001	CN54gp140 with GLA-AF	Env immunogens	I	2013.10–2015.11
CR104488/HIV-V-A003/IPCAVD008	Trimeric gp140 with/without aluminum phosphate	Env immunogens	I	2014.12–2016.04
FLSC-001	Full length single chain gp120-CD4 complex vaccine	Env immunogens	I	2015.11–2018.07
CR100965/HIV-V-A002/IPCAVD006	MVA Mosaic HIV in individuals with/without prior Ad26.ENVA.01	Mosaic vaccine	I	2014.09–2015.11
CR106152/HIV-V-A004/IPCAVD009	Ad26 Mosaic HIV prime, Ad26 Mosaic HIV or MVA Mosaic (*env* or *gag*-*pol*) and/or clade C gp140/aluminum phosphate boost	Mosaic vaccine	I/II	2014.12–2019.04
CR108152/VAC89220HPX2004	Ad26 Mosaic HIV or Ad26 Mosaic4 HIV prime (*env* or *gag*-*pol*), clade C gp140/aluminum phosphate and Ad26 Mosaic HIV or Ad26 Mosaic4 HIV boost	Mosaic vaccine	II	2016.07–2018.09
CR108068/VAC89220HPX1002	Ad26 Mosaic HIV (*env* or *gag*-*pol*) with clade C gp140/aluminum phosphate prime and boost	Mosaic vaccine	I	2016.03–2019.01
HVTN 090/NCT01438606	VSV-Indiana HIV *gag* vaccine	Replicating vectors	I	2011.10–2013.01
NCT01989533	Ad4-mgag and Ad4-*env*C150	Replicating vectors	I	2013.11–2020.02
HVTN 110	Ad4-mgag and/or Ad4-*env*C150 prime, AIDSVAX gp120 B/E/aluminum hydroxide boost	Replicating vectors	I	2015.03–2017.02
rcAd001/IAVI R001	RcAd26.Mosaic1.HIV-*env*	Replicating vectors	I	2015.01–2016.06
HVTN076/NCT00955006	VRC-HIVDNA-016-00-VP prime (clade B *gag*, *pol*, *nef*, clade ABC *env*) VRC-HIVADV014-00-VP boost (clade B *gag*-*pol* and clade ABC *env*)	DNA-based	I	2011.05–2013.09
HVTN 087	HIV-MAG vaccine with/without IL-12 pDNA adjuvant electroporation prime, VSV HIV *gag* boost	DNA-based	I	2012.05–2014.09
CRO2059	HIV DNA (CN54ENV/ZM6GPN) prime, MVA-/CN54rgp140/GLA-AF adjuvant boost	DNA based	I	2014–2016
HVTN 092	DNA-HIV-PT123 prime with/without NYVAC-HIV-PT1 and NYVAC-HIV-PT4 boost	DNA-based	I	2013.04–2014.09
HIV-CORE 004/IAVI N004	Ad35-GRIN/MVA.HIVconsv with/without pSG2. HIVconsv DNA with/without electroporation	DNA-based	I/II	2014.03–2015.08
HVTN 106	DNA Nat-B *env* or DNA CON-S *env* or DNA Mosaic *env* prime, MVA-CMDR boost	DNA-based	I	2015.01–2020.09
HVTN 098	PENNVAX^®^-GP HIV-1 DNA (*gag*, *pol*, *env*) vaccine with electroporation with/without IL-12 DNA adjuvant	DNA-based	I	2015.04–2016.08
CUTHIVAC002	HIV DNA-C CN54*env* prime with and without electroporation, CN54gp140 boost	DNA-based	I	2015.11–2017.04
VRI01	LIPO-5 or MVA HIV-B LIPO-5 or MVA HIV-B or GTU-Multi HIV B prime and LIPO-5 or MVA HIV-B boost	Lipopeptides	I/II	2014.03–2016.03

Some of the current ongoing and recently completed human clinical trials are shown. Note: This is not a complete list. Ad = Adenovirus; CN = Chinese; CUTHIVAC = Cutaneous and Mucosal HIV Vaccination; Env = viral envelope; FLSC = full-length single chain; GLA-AF = glucopyranosyl lipid adjuvant–aqueous formulation; GP = glycoprotein; HVTN = HIV Vaccine Trials Network; IAVI = International AIDS Vaccine Initiative; IPCAVD = Integrated Preclinical/Clinical AIDS Vaccine Development Program; MVA = modified vaccinia virus Ankara; NCT = National Clinical Trials identifier; vCP = canarypox vector; VRC = Vaccine Research Centre (USA); VRI = Vaccine Research Institute.

## References

[1] Joint United Nations Programme on HIV/AIDS (UNAIDS) UNAIDS DATA 2017. http://www.unaids.org/sites/default/files/media_asset/20170720_Data_book_2017_en.pdf.

[2] Yuan Z., Kang G., Ma F., Lu W., Fan W., Fennessey C.M., Keele B.F., Li Q. (2016). Recapitulating Cross-Species Transmission of Simian Immunodeficiency Virus SIVcpz to Humans by Using Humanized BLT Mice. J. Virol..

[3] Paraskevis D., Lemey P., Salemi M., Suchard M., Van De Peer Y., Vandamme A.M. (2003). Analysis of the evolutionary relationships of HIV-1 and SIVcpz sequences using bayesian inference: Implications for the origin of HIV-1. Mol. Biol. Evol..

[4] Sharp P.M., Hahn B.H. (2011). Origins of HIV and the AIDS pandemic. Cold Spring Harb. Perspect. Med..

[5] Cavrois M., Banerjee T., Mukherjee G., Raman N., Hussien R., Rodriguez B.A., Vasquez J., Spitzer M.H., Lazarus N.H., Jones J.J. (2017). Mass Cytometric Analysis of HIV Entry, Replication, and Remodeling in Tissue CD4+ T Cells. Cell Rep..

[6] Alimonti J.B., Ball T.B., Fowke K.R. (2003). Mechanisms of CD4+ T lymphocyte cell death in human immunodeficiency virus infection and AIDS. J. Gen. Virol..

[7] Finkel T.H., Tudor-Williams G., Banda N.K., Cotton M.F., Curiel T., Monks C., Baba T.W., Ruprecht R.M., Kupfer A. (1995). Apoptosis occurs predominantly in bystander cells and not in productively infected cells of HIV- and SIV-infected lymph nodes. Nat. Med..

[8] Doitsh G., Galloway N.L., Geng X., Yang Z., Monroe K.M., Zepeda O., Hunt P.W., Hatano H., Sowinski S., Munoz-Arias I. (2014). Cell death by pyroptosis drives CD4 T-cell depletion in HIV-1 infection. Nature.

[9] Munoz-Arias I., Doitsh G., Yang Z., Sowinski S., Ruelas D., Greene W.C. (2015). Blood-Derived CD4 T Cells Naturally Resist Pyroptosis during Abortive HIV-1 Infection. Cell Host Microbe.

[10] Pantaleo G., Graziosi C., Butini L., Pizzo P.A., Schnittman S.M., Kotler D.P., Fauci A.S. (1991). Lymphoid organs function as major reservoirs for human immunodeficiency virus. Proc. Natl. Acad. Sci. USA.

[11] Abrahams M.R., Anderson J.A., Giorgi E.E., Seoighe C., Mlisana K., Ping L.H., Athreya G.S., Treurnicht F.K., Keele B.F., Wood N. (2009). Quantitating the multiplicity of infection with human immunodeficiency virus type 1 subtype C reveals a non-poisson distribution of transmitted variants. J. Virol..

[12] Keele B.F., Giorgi E.E., Salazar-Gonzalez J.F., Decker J.M., Pham K.T., Salazar M.G., Sun C., Grayson T., Wang S., Li H. (2008). Identification and characterization of transmitted and early founder virus envelopes in primary HIV-1 infection. Proc. Natl. Acad. Sci. USA.

[13] Hu J., Gardner M.B., Miller C.J. (2000). Simian immunodeficiency virus rapidly penetrates the cervicovaginal mucosa after intravaginal inoculation and infects intraepithelial dendritic cells. J. Virol..

[14] Miller C.J., Hu J. (1999). T cell-tropic simian immunodeficiency virus (SIV) and simian-human immunodeficiency viruses are readily transmitted by vaginal inoculation of rhesus macaques, and Langerhans’ cells of the female genital tract are infected with SIV. J. Infect. Dis..

[15] Spira A.I., Marx P.A., Patterson B.K., Mahoney J., Koup R.A., Wolinsky S.M., Ho D.D. (1996). Cellular targets of infection and route of viral dissemination after an intravaginal inoculation of simian immunodeficiency virus into rhesus macaques. J. Exp. Med..

[16] Zhang Z., Schuler T., Zupancic M., Wietgrefe S., Staskus K.A., Reimann K.A., Reinhart T.A., Rogan M., Cavert W., Miller C.J. (1999). Sexual transmission and propagation of SIV and HIV in resting and activated CD4+ T cells. Science.

[17] Li Q., Estes J.D., Schlievert P.M., Duan L., Brosnahan A.J., Southern P.J., Reilly C.S., Peterson M.L., Schultz-Darken N., Brunner K.G. (2009). Glycerol monolaurate prevents mucosal SIV transmission. Nature.

[18] Ballweber L., Robinson B., Kreger A., Fialkow M., Lentz G., McElrath M.J., Hladik F. (2011). Vaginal langerhans cells nonproductively transporting HIV-1 mediate infection of T cells. J. Virol..

[19] Mohammed J., Beura L.K., Bobr A., Astry B., Chicoine B., Kashem S.W., Welty N.E., Igyarto B.Z., Wijeyesinghe S., Thompson E.A. (2016). Stromal cells control the epithelial residence of DCs and memory T cells by regulated activation of TGF-beta. Nat. Immunol..

[20] Rodriguez-Garcia M., Shen Z., Barr F.D., Boesch A.W., Ackerman M.E., Kappes J.C., Ochsenbauer C., Wira C.R. (2017). Dendritic cells from the human female reproductive tract rapidly capture and respond to HIV. Mucosal Immunol..

[21] Stieh D.J., Matias E., Xu H., Fought A.J., Blanchard J.L., Marx P.A., Veazey R.S., Hope T.J. (2016). Th17 Cells Are Preferentially Infected Very Early after Vaginal Transmission of SIV in Macaques. Cell Host Microbe.

[22] Pudney J., Quayle A.J., Anderson D.J. (2005). Immunological microenvironments in the human vagina and cervix: Mediators of cellular immunity are concentrated in the cervical transformation zone. Biol. Reprod..

[23] Haase A.T. (2010). Targeting early infection to prevent HIV-1 mucosal transmission. Nature.

[24] Whitney J.B., Hill A.L., Sanisetty S., Penaloza-MacMaster P., Liu J., Shetty M., Parenteau L., Cabral C., Shields J., Blackmore S. (2014). Rapid seeding of the viral reservoir prior to SIV viraemia in rhesus monkeys. Nature.

[25] Goonetilleke N., Liu M.K., Salazar-Gonzalez J.F., Ferrari G., Giorgi E., Ganusov V.V., Keele B.F., Learn G.H., Turnbull E.L., Salazar M.G. (2009). The first T cell response to transmitted/founder virus contributes to the control of acute viremia in HIV-1 infection. J. Exp. Med..

[26] Buckner C.M., Moir S., Ho J., Wang W., Posada J.G., Kardava L., Funk E.K., Nelson A.K., Li Y., Chun T.W. (2013). Characterization of plasmablasts in the blood of HIV-infected viremic individuals: Evidence for nonspecific immune activation. J. Virol..

[27] De Silva N.S., Klein U. (2015). Dynamics of B cells in germinal centres. Nat. Rev. Immunol..

[28] Borrow P., Moody M.A. (2017). Immunologic characteristics of HIV-infected individuals who make broadly neutralizing antibodies. Immunol. Rev..

[29] Petrovas C., Yamamoto T., Gerner M.Y., Boswell K.L., Wloka K., Smith E.C., Ambrozak D.R., Sandler N.G., Timmer K.J., Sun X. (2012). CD4 T follicular helper cell dynamics during SIV infection. J. Clin. Investig..

[30] Yamamoto T., Lynch R.M., Gautam R., Matus-Nicodemos R., Schmidt S.D., Boswell K.L., Darko S., Wong P., Sheng Z., Petrovas C. (2015). Quality and quantity of TFH cells are critical for broad antibody development in SHIVAD8 infection. Sci. Transl. Med..

[31] Tomaras G.D., Yates N.L., Liu P., Qin L., Fouda G.G., Chavez L.L., Decamp A.C., Parks R.J., Ashley V.C., Lucas J.T. (2008). Initial B-cell responses to transmitted human immunodeficiency virus type 1: Virion-binding immunoglobulin M (IgM) and IgG antibodies followed by plasma anti-gp41 antibodies with ineffective control of initial viremia. J. Virol..

[32] Liao H.X., Chen X., Munshaw S., Zhang R., Marshall D.J., Vandergrift N., Whitesides J.F., Lu X., Yu J.S., Hwang K.K. (2011). Initial antibodies binding to HIV-1 gp41 in acutely infected subjects are polyreactive and highly mutated. J. Exp. Med..

[33] Trama A.M., Moody M.A., Alam S.M., Jaeger F.H., Lockwood B., Parks R., Lloyd K.E., Stolarchuk C., Scearce R., Foulger A. (2014). HIV-1 envelope gp41 antibodies can originate from terminal ileum B cells that share cross-reactivity with commensal bacteria. Cell Host Microbe.

[34] Yates N.L., Stacey A.R., Nolen T.L., Vandergrift N.A., Moody M.A., Montefiori D.C., Weinhold K.J., Blattner W.A., Borrow P., Shattock R. (2013). HIV-1 gp41 envelope IgA is frequently elicited after transmission but has an initial short response half-life. Mucosal Immunol..

[35] Kulkarni V., Ruprecht R.M. (2017). Mucosal IgA Responses: Damaged in Established HIV Infection-Yet, Effective Weapon against HIV Transmission. Front. Immunol..

[36] Wei X., Decker J.M., Wang S., Hui H., Kappes J.C., Wu X., Salazar-Gonzalez J.F., Salazar M.G., Kilby J.M., Saag M.S. (2003). Antibody neutralization and escape by HIV-1. Nature.

[37] Richman D.D., Wrin T., Little S.J., Petropoulos C.J. (2003). Rapid evolution of the neutralizing antibody response to HIV type 1 infection. Proc. Natl. Acad. Sci. USA.

[38] Liao H.X., Lynch R., Zhou T., Gao F., Alam S.M., Boyd S.D., Fire A.Z., Roskin K.M., Schramm C.A., Zhang Z. (2013). Co-evolution of a broadly neutralizing HIV-1 antibody and founder virus. Nature.

[39] Tomaras G.D., Binley J.M., Gray E.S., Crooks E.T., Osawa K., Moore P.L., Tumba N., Tong T., Shen X., Yates N.L. (2011). Polyclonal B cell responses to conserved neutralization epitopes in a subset of HIV-1-infected individuals. J. Virol..

[40] Walker L.M., Huber M., Doores K.J., Falkowska E., Pejchal R., Julien J.P., Wang S.K., Ramos A., Chan-Hui P.Y., Moyle M. (2011). Broad neutralization coverage of HIV by multiple highly potent antibodies. Nature.

[41] Doria-Rose N.A., Klein R.M., Daniels M.G., O’Dell S., Nason M., Lapedes A., Bhattacharya T., Migueles S.A., Wyatt R.T., Korber B.T. (2010). Breadth of human immunodeficiency virus-specific neutralizing activity in sera: Clustering analysis and association with clinical variables. J. Virol..

[42] Bonsignori M., Montefiori D.C., Wu X., Chen X., Hwang K.K., Tsao C.Y., Kozink D.M., Parks R.J., Tomaras G.D., Crump J.A. (2012). Two distinct broadly neutralizing antibody specificities of different clonal lineages in a single HIV-1-infected donor: Implications for vaccine design. J. Virol..

[43] Walker L.M., Simek M.D., Priddy F., Gach J.S., Wagner D., Zwick M.B., Phogat S.K., Poignard P., Burton D.R. (2010). A limited number of antibody specificities mediate broad and potent serum neutralization in selected HIV-1 infected individuals. PLoS Pathog..

[44] Baba T.W., Liska V., Hofmann-Lehmann R., Vlasak J., Xu W., Ayehunie S., Cavacini L.A., Posner M.R., Katinger H., Stiegler G. (2000). Human neutralizing monoclonal antibodies of the IgG1 subtype protect against mucosal simian-human immunodeficiency virus infection. Nat. Med..

[45] Hessell A.J., Rakasz E.G., Poignard P., Hangartner L., Landucci G., Forthal D.N., Koff W.C., Watkins D.I., Burton D.R. (2009). Broadly neutralizing human anti-HIV antibody 2G12 is effective in protection against mucosal SHIV challenge even at low serum neutralizing titers. PLoS Pathog..

[46] Klein K., Veazey R.S., Warrier R., Hraber P., Doyle-Meyers L.A., Buffa V., Liao H.X., Haynes B.F., Shaw G.M., Shattock R.J. (2013). Neutralizing IgG at the portal of infection mediates protection against vaginal simian/human immunodeficiency virus challenge. J. Virol..

[47] Mascola J.R., Lewis M.G., Stiegler G., Harris D., VanCott T.C., Hayes D., Louder M.K., Brown C.R., Sapan C.V., Frankel S.S. (1999). Protection of Macaques against pathogenic simian/human immunodeficiency virus 89.6PD by passive transfer of neutralizing antibodies. J. Virol..

[48] Parren P.W., Marx P.A., Hessell A.J., Luckay A., Harouse J., Cheng-Mayer C., Moore J.P., Burton D.R. (2001). Antibody protects macaques against vaginal challenge with a pathogenic R5 simian/human immunodeficiency virus at serum levels giving complete neutralization in vitro. J. Virol..

[49] Xu W., Hofmann-Lehmann R., McClure H.M., Ruprecht R.M. (2002). Passive immunization with human neutralizing monoclonal antibodies: Correlates of protective immunity against HIV. Vaccine.

[50] Yang G., Holl T.M., Liu Y., Li Y., Lu X., Nicely N.I., Kepler T.B., Alam S.M., Liao H.X., Cain D.W. (2013). Identification of autoantigens recognized by the 2F5 and 4E10 broadly neutralizing HIV-1 antibodies. J. Exp. Med..

[51] Liu M., Yang G., Wiehe K., Nicely N.I., Vandergrift N.A., Rountree W., Bonsignori M., Alam S.M., Gao J., Haynes B.F. (2015). Polyreactivity and autoreactivity among HIV-1 antibodies. J. Virol..

[52] Bonsignori M., Wiehe K., Grimm S.K., Lynch R., Yang G., Kozink D.M., Perrin F., Cooper A.J., Hwang K.K., Chen X. (2014). An autoreactive antibody from an SLE/HIV-1 individual broadly neutralizes HIV-1. J. Clin. Investig..

[53] Haynes B.F., Fleming J., St Clair E.W., Katinger H., Stiegler G., Kunert R., Robinson J., Scearce R.M., Plonk K., Staats H.F. (2005). Cardiolipin polyspecific autoreactivity in two broadly neutralizing HIV-1 antibodies. Science.

[54] Meffre E., Milili M., Blanco-Betancourt C., Antunes H., Nussenzweig M.C., Schiff C. (2001). Immunoglobulin heavy chain expression shapes the B cell receptor repertoire in human B cell development. J. Clin. Investig..

[55] Meffre E., Wardemann H. (2008). B-cell tolerance checkpoints in health and autoimmunity. Curr. Opin. Immunol..

[56] Wu X., Zhang Z., Schramm C.A., Joyce M.G., Kwon Y.D., Zhou T., Sheng Z., Zhang B., O’Dell S., McKee K. (2015). Maturation and Diversity of the VRC01-Antibody Lineage over 15 Years of Chronic HIV-1 Infection. Cell.

[57] Berman P.W., Gregory T.J., Riddle L., Nakamura G.R., Champe M.A., Porter J.P., Wurm F.M., Hershberg R.D., Cobb E.K., Eichberg J.W. (1990). Protection of chimpanzees from infection by HIV-1 after vaccination with recombinant glycoprotein gp120 but not gp160. Nature.

[58] El-Amad Z., Murthy K.K., Higgins K., Cobb E.K., Haigwood N.L., Levy J.A., Steimer K.S. (1995). Resistance of chimpanzees immunized with recombinant gp120SF2 to challenge by HIV-1SF2. AIDS.

[59] Berman P.W., Murthy K.K., Wrin T., Vennari J.C., Cobb E.K., Eastman D.J., Champe M., Nakamura G.R., Davison D., Powell M.F. (1996). Protection of MN-rgp120-immunized chimpanzees from heterologous infection with a primary isolate of human immunodeficiency virus type 1. J. Infect. Dis..

[60] Belshe R.B., Clements M.L., Dolin R., Graham B.S., McElrath J., Gorse G.J., Schwartz D., Keefer M.C., Wright P., Corey L. (1993). Safety and immunogenicity of a fully glycosylated recombinant gp160 human immunodeficiency virus type 1 vaccine in subjects at low risk of infection. National Institute of Allergy and Infectious Diseases AIDS Vaccine Evaluation Group Network. J. Infect. Dis..

[61] Keefer M.C., Graham B.S., Belshe R.B., Schwartz D., Corey L., Bolognesi D.P., Stablein D.M., Montefiori D.C., McElrath M.J., Clements M.L. (1994). Studies of high doses of a human immunodeficiency virus type 1 recombinant glycoprotein 160 candidate vaccine in HIV type 1-seronegative humans. The AIDS Vaccine Clinical Trials Network. AIDS Res. Hum. Retrovir..

[62] Gorse G.J., Rogers J.H., Perry J.E., Newman F.K., Frey S.E., Patel G.B., Belshe R.B. (1995). HIV-1 recombinant gp160 vaccine induced antibodies in serum and saliva. The NIAID AIDS Vaccine Clinical Trials Network. Vaccine.

[63] Migasena S., Suntharasamai P., Pitisuttithum P., Kitayaporn D., Wasi C., Huang W., Vanichseni S., Koompong C., Kaewkungwal J., Raktham S. (2000). AIDSVAX (MN) in Bangkok injecting drug users: A report on safety and immunogenicity, including macrophage-tropic virus neutralization. AIDS Res. Hum. Retrovir..

[64] Pitisuttithum P., Nitayaphan S., Thongcharoen P., Khamboonruang C., Kim J., de Souza M., Chuenchitra T., Garner R.P., Thapinta D., Polonis V. (2003). Safety and immunogenicity of combinations of recombinant subtype E and B human immunodeficiency virus type 1 envelope glycoprotein 120 vaccines in healthy Thai adults. J. Infect. Dis..

[65] Mooij P., van der Kolk M., Bogers W.M., ten Haaft P.J., Van Der Meide P., Almond N., Stott J., Deschamps M., Labbe D., Momin P. (1998). A clinically relevant HIV-1 subunit vaccine protects rhesus macaques from in vivo passaged simian-human immunodeficiency virus infection. AIDS.

[66] Stott E.J., Almond N., Kent K., Walker B., Hull R., Rose J., Silvera P., Sangster R., Corcoran T., Lines J. (1998). Evaluation of a candidate human immunodeficiency virus type 1 (HIV-1) vaccine in macaques: Effect of vaccination with HIV-1 gp120 on subsequent challenge with heterologous simian immunodeficiency virus-HIV-1 chimeric virus. J. Gen. Virol..

[67] Pitisuttithum P., Gilbert P., Gurwith M., Heyward W., Martin M., van Griensven F., Hu D., Tappero J.W., Choopanya K., Bangkok Vaccine Evaluation G. (2006). Randomized, double-blind, placebo-controlled efficacy trial of a bivalent recombinant glycoprotein 120 HIV-1 vaccine among injection drug users in Bangkok, Thailand. J. Infect. Dis..

[68] Flynn N.M., Forthal D.N., Harro C.D., Judson F.N., Mayer K.H., Para M.F., rgp120 HIV Vaccine Study Group (2005). Placebo-controlled phase 3 trial of a recombinant glycoprotein 120 vaccine to prevent HIV-1 infection. J. Infect. Dis..

[69] Emu B., Sinclair E., Hatano H., Ferre A., Shacklett B., Martin J.N., McCune J.M., Deeks S.G. (2008). HLA class I-restricted T-cell responses may contribute to the control of human immunodeficiency virus infection, but such responses are not always necessary for long-term virus control. J. Virol..

[70] Altfeld M., Kalife E.T., Qi Y., Streeck H., Lichterfeld M., Johnston M.N., Burgett N., Swartz M.E., Yang A., Alter G. (2006). HLA Alleles Associated with Delayed Progression to AIDS Contribute Strongly to the Initial CD8(+) T Cell Response against HIV-1. PLoS Med..

[71] Harrer T., Harrer E., Kalams S.A., Elbeik T., Staprans S.I., Feinberg M.B., Cao Y., Ho D.D., Yilma T., Caliendo A.M. (1996). Strong cytotoxic T cell and weak neutralizing antibody responses in a subset of persons with stable nonprogressing HIV type 1 infection. AIDS Res. Hum. Retrovir..

[72] Jin X., Bauer D.E., Tuttleton S.E., Lewin S., Gettie A., Blanchard J., Irwin C.E., Safrit J.T., Mittler J., Weinberger L. (1999). Dramatic rise in plasma viremia after CD8(+) T cell depletion in simian immunodeficiency virus-infected macaques. J. Exp. Med..

[73] Schmitz J.E., Kuroda M.J., Santra S., Sasseville V.G., Simon M.A., Lifton M.A., Racz P., Tenner-Racz K., Dalesandro M., Scallon B.J. (1999). Control of viremia in simian immunodeficiency virus infection by CD8^+^ lymphocytes. Science.

[74] Shiver J.W., Fu T.M., Chen L., Casimiro D.R., Davies M.E., Evans R.K., Zhang Z.Q., Simon A.J., Trigona W.L., Dubey S.A. (2002). Replication-incompetent adenoviral vaccine vector elicits effective anti-immunodeficiency-virus immunity. Nature.

[75] Buchbinder S.P., Mehrotra D.V., Duerr A., Fitzgerald D.W., Mogg R., Li D., Gilbert P.B., Lama J.R., Marmor M., Del Rio C. (2008). Efficacy assessment of a cell-mediated immunity HIV-1 vaccine (the Step Study): A double-blind, randomised, placebo-controlled, test-of-concept trial. Lancet.

[76] Gray G.E., Allen M., Moodie Z., Churchyard G., Bekker L.G., Nchabeleng M., Mlisana K., Metch B., de Bruyn G., Latka M.H. (2011). Safety and efficacy of the HVTN 503/Phambili study of a clade-B-based HIV-1 vaccine in South Africa: A double-blind, randomised, placebo-controlled test-of-concept phase 2b study. Lancet Infect. Dis..

[77] Rolland M., Tovanabutra S., deCamp A.C., Frahm N., Gilbert P.B., Sanders-Buell E., Heath L., Magaret C.A., Bose M., Bradfield A. (2011). Genetic impact of vaccination on breakthrough HIV-1 sequences from the STEP trial. Nat. Med..

[78] Rerks-Ngarm S., Pitisuttithum P., Nitayaphan S., Kaewkungwal J., Chiu J., Paris R., Premsri N., Namwat C., de Souza M., Adams E. (2009). Vaccination with ALVAC and AIDSVAX to prevent HIV-1 infection in Thailand. N. Engl. J. Med..

[79] Robb M.L., Rerks-Ngarm S., Nitayaphan S., Pitisuttithum P., Kaewkungwal J., Kunasol P., Khamboonruang C., Thongcharoen P., Morgan P., Benenson M. (2012). Risk behaviour and time as covariates for efficacy of the HIV vaccine regimen ALVAC-HIV (vCP1521) and AIDSVAX B/E: A post-hoc analysis of the Thai phase 3 efficacy trial RV 144. Lancet Infect. Dis..

[80] Gartland A.J., Li S., McNevin J., Tomaras G.D., Gottardo R., Janes H., Fong Y., Morris D., Geraghty D.E., Kijak G.H. (2014). Analysis of HLA A*02 association with vaccine efficacy in the RV144 HIV-1 vaccine trial. J. Virol..

[81] Liao H.X., Bonsignori M., Alam S.M., McLellan J.S., Tomaras G.D., Moody M.A., Kozink D.M., Hwang K.K., Chen X., Tsao C.Y. (2013). Vaccine induction of antibodies against a structurally heterogeneous site of immune pressure within HIV-1 envelope protein variable regions 1 and 2. Immunity.

[82] Hammer S.M., Sobieszczyk M.E., Janes H., Karuna S.T., Mulligan M.J., Grove D., Koblin B.A., Buchbinder S.P., Keefer M.C., Tomaras G.D. (2013). Efficacy trial of a DNA/rAd5 HIV-1 preventive vaccine. N. Engl. J. Med..

[83] DeCamp A.C., Rolland M., Edlefsen P.T., Sanders-Buell E., Hall B., Magaret C.A., Fiore-Gartland A.J., Juraska M., Carpp L.N., Karuna S.T. (2017). Sieve analysis of breakthrough HIV-1 sequences in HVTN 505 identifies vaccine pressure targeting the CD4 binding site of Env-gp120. PLoS ONE.

[84] Baba T.W., Liska V., Khimani A.H., Ray N.B., Dailey P.J., Penninck D., Bronson R., Greene M.F., McClure H.M., Martin L.N. (1999). Live attenuated, multiply deleted simian immunodeficiency virus causes AIDS in infant and adult macaques. Nat. Med..

[85] Daniel M.D., Kirchhoff F., Czajak S.C., Sehgal P.K., Desrosiers R.C. (1992). Protective effects of a live attenuated SIV vaccine with a deletion in the nef gene. Science.

[86] Learmont J., Tindall B., Evans L., Cunningham A., Cunningham P., Wells J., Penny R., Kaldor J., Cooper D.A. (1992). Long-term symptomless HIV-1 infection in recipients of blood products from a single donor. Lancet.

[87] Mikell I., Sather D.N., Kalams S.A., Altfeld M., Alter G., Stamatatos L. (2011). Characteristics of the earliest cross-neutralizing antibody response to HIV-1. PLoS Pathog..

[88] Van Gils M.J., Sanders R.W. (2013). Broadly neutralizing antibodies against HIV-1: Templates for a vaccine. Virology.

[89] Poignard P., Moulard M., Golez E., Vivona V., Franti M., Venturini S., Wang M., Parren P.W., Burton D.R. (2003). Heterogeneity of envelope molecules expressed on primary human immunodeficiency virus type 1 particles as probed by the binding of neutralizing and nonneutralizing antibodies. J. Virol..

[90] McCoy L.E., van Gils M.J., Ozorowski G., Messmer T., Briney B., Voss J.E., Kulp D.W., Macauley M.S., Sok D., Pauthner M. (2016). Holes in the Glycan Shield of the Native HIV Envelope Are a Target of Trimer-Elicited Neutralizing Antibodies. Cell Rep..

[91] Kwon Y.D., Pancera M., Acharya P., Georgiev I.S., Crooks E.T., Gorman J., Joyce M.G., Guttman M., Ma X., Narpala S. (2015). Crystal structure, conformational fixation and entry-related interactions of mature ligand-free HIV-1 Env. Nat. Struct. Mol. Biol..

[92] Berman H.M., Westbrook J., Feng Z., Gilliland G., Bhat T.N., Weissig H., Shindyalov I.N., Bourne P.E. (2000). The Protein Data Bank. Nucleic Acids Res..

[93] Pettersen E.F., Goddard T.D., Huang C.C., Couch G.S., Greenblatt D.M., Meng E.C., Ferrin T.E. (2004). UCSF Chimera—A visualization system for exploratory research and analysis. J. Comput. Chem..

[94] Xiao X., Chen W., Feng Y., Zhu Z., Prabakaran P., Wang Y., Zhang M.Y., Longo N.S., Dimitrov D.S. (2009). Germline-like predecessors of broadly neutralizing antibodies lack measurable binding to HIV-1 envelope glycoproteins: Implications for evasion of immune responses and design of vaccine immunogens. Biochem. Biophys. Res. Commun..

[95] Doria-Rose N.A., Schramm C.A., Gorman J., Moore P.L., Bhiman J.N., DeKosky B.J., Ernandes M.J., Georgiev I.S., Kim H.J., Pancera M. (2014). Developmental pathway for potent V1V2-directed HIV-neutralizing antibodies. Nature.

[96] Scheid J.F., Mouquet H., Feldhahn N., Seaman M.S., Velinzon K., Pietzsch J., Ott R.G., Anthony R.M., Zebroski H., Hurley A. (2009). Broad diversity of neutralizing antibodies isolated from memory B cells in HIV-infected individuals. Nature.

[97] Kepler T.B., Liao H.X., Alam S.M., Bhaskarabhatla R., Zhang R., Yandava C., Stewart S., Anasti K., Kelsoe G., Parks R. (2014). Immunoglobulin gene insertions and deletions in the affinity maturation of HIV-1 broadly reactive neutralizing antibodies. Cell Host Microbe.

[98] Escolano A., Steichen J.M., Dosenovic P., Kulp D.W., Golijanin J., Sok D., Freund N.T., Gitlin A.D., Oliveira T., Araki T. (2016). Sequential Immunization Elicits Broadly Neutralizing Anti-HIV-1 Antibodies in Ig Knockin Mice. Cell.

[99] Steichen J.M., Kulp D.W., Tokatlian T., Escolano A., Dosenovic P., Stanfield R.L., McCoy L.E., Ozorowski G., Hu X., Kalyuzhniy O. (2016). HIV Vaccine Design to Target Germline Precursors of Glycan-Dependent Broadly Neutralizing Antibodies. Immunity.

[100] Landais E., Murrell B., Briney B., Murrell S., Rantalainen K., Berndsen Z.T., Ramos A., Wickramasinghe L., Smith M.L., Eren K. (2017). HIV Envelope Glycoform Heterogeneity and Localized Diversity Govern the Initiation and Maturation of a V2 Apex Broadly Neutralizing Antibody Lineage. Immunity.

[101] Abbott R.K., Lee J.H., Menis S., Skog P., Rossi M., Ota T., Kulp D.W., Bhullar D., Kalyuzhniy O., Havenar-Daughton C. (2018). Precursor Frequency and Affinity Determine B Cell Competitive Fitness in Germinal Centers, Tested with Germline-Targeting HIV Vaccine Immunogens. Immunity.

[102] Derdeyn C.A., Decker J.M., Bibollet-Ruche F., Mokili J.L., Muldoon M., Denham S.A., Heil M.L., Kasolo F., Musonda R., Hahn B.H. (2004). Envelope-constrained neutralization-sensitive HIV-1 after heterosexual transmission. Science.

[103] Chohan B., Lang D., Sagar M., Korber B., Lavreys L., Richardson B., Overbaugh J. (2005). Selection for human immunodeficiency virus type 1 envelope glycosylation variants with shorter V1-V2 loop sequences occurs during transmission of certain genetic subtypes and may impact viral RNA levels. J. Virol..

[104] Li B., Decker J.M., Johnson R.W., Bibollet-Ruche F., Wei X., Mulenga J., Allen S., Hunter E., Hahn B.H., Shaw G.M. (2006). Evidence for potent autologous neutralizing antibody titers and compact envelopes in early infection with subtype C human immunodeficiency virus type 1. J. Virol..

[105] Klasse P.J., Depetris R.S., Pejchal R., Julien J.P., Khayat R., Lee J.H., Marozsan A.J., Cupo A., Cocco N., Korzun J. (2013). Influences on trimerization and aggregation of soluble, cleaved HIV-1 SOSIP envelope glycoprotein. J. Virol..

[106] Cheng W. (2016). The Density Code for the Development of a Vaccine?. J. Pharm. Sci..

[107] Yang X., Kurteva S., Ren X., Lee S., Sodroski J. (2005). Stoichiometry of envelope glycoprotein trimers in the entry of human immunodeficiency virus type 1. J. Virol..

[108] Klasse P.J. (2007). Modeling how many envelope glycoprotein trimers per virion participate in human immunodeficiency virus infectivity and its neutralization by antibody. Virology.

[109] Magnus C., Rusert P., Bonhoeffer S., Trkola A., Regoes R.R. (2009). Estimating the stoichiometry of human immunodeficiency virus entry. J. Virol..

[110] Brandenberg O.F., Magnus C., Rusert P., Regoes R.R., Trkola A. (2015). Different infectivity of HIV-1 strains is linked to number of envelope trimers required for entry. PLoS Pathog..

[111] Gnanakaran S., Bhattacharya T., Daniels M., Keele B.F., Hraber P.T., Lapedes A.S., Shen T., Gaschen B., Krishnamoorthy M., Li H. (2011). Recurrent signature patterns in HIV-1 B clade envelope glycoproteins associated with either early or chronic infections. PLoS Pathog..

[112] Li Y., Luo L., Thomas D.Y., Kang C.Y. (2000). The HIV-1 Env protein signal sequence retards its cleavage and down-regulates the glycoprotein folding. Virology.

[113] Li Y., Bergeron J.J., Luo L., Ou W.J., Thomas D.Y., Kang C.Y. (1996). Effects of inefficient cleavage of the signal sequence of HIV-1 gp 120 on its association with calnexin, folding, and intracellular transport. Proc. Natl. Acad. Sci. USA.

[114] Asmal M., Hellmann I., Liu W., Keele B.F., Perelson A.S., Bhattacharya T., Gnanakaran S., Daniels M., Haynes B.F., Korber B.T. (2011). A signature in HIV-1 envelope leader peptide associated with transition from acute to chronic infection impacts envelope processing and infectivity. PLoS ONE.

[115] Parrish N.F., Gao F., Li H., Giorgi E.E., Barbian H.J., Parrish E.H., Zajic L., Iyer S.S., Decker J.M., Kumar A. (2013). Phenotypic properties of transmitted founder HIV-1. Proc. Natl. Acad. Sci. USA.

[116] McCurley N.P., Domi A., Basu R., Saunders K.O., LaBranche C.C., Montefiori D.C., Haynes B.F., Robinson H.L. (2017). HIV transmitted/founder vaccines elicit autologous tier 2 neutralizing antibodies for the CD4 binding site. PLoS ONE.

[117] Bonsignori M., Kreider E.F., Fera D., Meyerhoff R.R., Bradley T., Wiehe K., Alam S.M., Aussedat B., Walkowicz W.E., Hwang K.K. (2017). Staged induction of HIV-1 glycan-dependent broadly neutralizing antibodies. Sci. Transl. Med..

[118] Sagar M., Wu X., Lee S., Overbaugh J. (2006). Human immunodeficiency virus type 1 V1-V2 envelope loop sequences expand and add glycosylation sites over the course of infection, and these modifications affect antibody neutralization sensitivity. J. Virol..

[119] Bradley T., Fera D., Bhiman J., Eslamizar L., Lu X., Anasti K., Zhang R., Sutherland L.L., Scearce R.M., Bowman C.M. (2016). Structural Constraints of Vaccine-Induced Tier-2 Autologous HIV Neutralizing Antibodies Targeting the Receptor-Binding Site. Cell Rep..

[120] Maxfield L.F., Abbink P., Stephenson K.E., Borducchi E.N., Kirilova M.M., Paulino N., Boyd M., Shabram P., Ruan Q., Patel M. (2015). Attenuation of Replication-Competent Adenovirus Serotype 26 Vaccines by Vectorization. Clin. Vaccine Immunol..

[121] Penaloza-MacMaster P., Provine N.M., Ra J., Borducchi E.N., McNally A., Simmons N.L., Iampietro M.J., Barouch D.H. (2013). Alternative serotype adenovirus vaccine vectors elicit memory T cells with enhanced anamnestic capacity compared to Ad5 vectors. J. Virol..

[122] Teigler J.E., Phogat S., Franchini G., Hirsch V.M., Michael N.L., Barouch D.H. (2014). The canarypox virus vector ALVAC induces distinct cytokine responses compared to the vaccinia virus-based vectors MVA and NYVAC in rhesus monkeys. J. Virol..

[123] Alexander J., Mendy J., Vang L., Avanzini J.B., Garduno F., Manayani D.J., Ishioka G., Farness P., Ping L.H., Swanstrom R. (2013). Pre-clinical development of a recombinant, replication-competent adenovirus serotype 4 vector vaccine expressing HIV-1 envelope 1086 clade C. PLoS ONE.

[124] Alayo Q.A., Provine N.M., Penaloza-MacMaster P. (2017). Novel Concepts for HIV Vaccine Vector Design. mSphere.

[125] Kim J.H., Excler J.L., Michael N.L. (2015). Lessons from the RV144 Thai phase III HIV-1 vaccine trial and the search for correlates of protection. Annu. Rev. Med..

[126] Barouch D.H., Stephenson K.E., Borducchi E.N., Smith K., Stanley K., McNally A.G., Liu J., Abbink P., Maxfield L.F., Seaman M.S. (2013). Protective efficacy of a global HIV-1 mosaic vaccine against heterologous SHIV challenges in rhesus monkeys. Cell.

[127] Experimental HIV Vaccine Regimen Is Well-Tolerated, Elicits Immune Responses. https://www.niaid.nih.gov/news-events/experimental-hiv-vaccine-regimen-well-tolerated-elicits-immune-responses.

[128] Safety, Tolerability, and Immunogenicity Study of Homologous Ad26 Mosaic Vector Vaccine Regimens or Heterologous Ad26 Mosaic and MVA Mosaic Vector Vaccine Regimens With Glycoprotein 140 (gp140) for Human Immunodeficiency Virus (HIV) Prevention. https://clinicaltrials.gov/ct2/show/NCT02315703.

[129] Safety, Tolerability and Immunogenicity Study of Different Vaccine Regimens of Trivalent Ad26.Mos.HIV or Tetravalent Ad26.Mos4.HIV Along With Clade C Glycoprotein (gp)140 in Healthy Human Immunodeficiency Virus (HIV)-Uninfected Adults. https://clinicaltrials.gov/ct2/show/NCT02788045.

[130] HPX2003/HVTN118/IPCAVD012/ASCENT. https://www.avac.org/trial/hpx2003hvtn118ipcavd012ascent.

[131] Ackerman M.E., Barouch D.H., Alter G. (2017). Systems serology for evaluation of HIV vaccine trials. Immunol. Rev..

[132] Tarca A.L., Carey V.J., Chen X.W., Romero R., Draghici S. (2007). Machine learning and its applications to biology. PLoS Comput. Biol..

[133] Nakaya H.I., Li S., Pulendran B. (2012). Systems vaccinology: Learning to compute the behavior of vaccine induced immunity. Wiley Interdiscip. Rev. Syst. Biol. Med..

[134] Arnold K.B., Chung A.W. (2018). Prospects from systems serology research. Immunology.

[135] Querec T.D., Akondy R.S., Lee E.K., Cao W., Nakaya H.I., Teuwen D., Pirani A., Gernert K., Deng J., Marzolf B. (2009). Systems biology approach predicts immunogenicity of the yellow fever vaccine in humans. Nat. Immunol..

[136] Nakaya H.I., Wrammert J., Lee E.K., Racioppi L., Marie-Kunze S., Haining W.N., Means A.R., Kasturi S.P., Khan N., Li G.M. (2011). Systems biology of vaccination for seasonal influenza in humans. Nat. Immunol..

[137] Vahey M.T., Wang Z., Kester K.E., Cummings J., Heppner D.G., Nau M.E., Ofori-Anyinam O., Cohen J., Coche T., Ballou W.R. (2010). Expression of genes associated with immunoproteasome processing of major histocompatibility complex peptides is indicative of protection with adjuvanted RTS,S malaria vaccine. J. Infect. Dis..

[138] Kazmin D., Nakaya H.I., Lee E.K., Johnson M.J., van der Most R., van den Berg R.A., Ballou W.R., Jongert E., Wille-Reece U., Ockenhouse C. (2017). Systems analysis of protective immune responses to RTS,S malaria vaccination in humans. Proc. Natl. Acad. Sci. USA.

[139] Churchyard G.J., Morgan C., Adams E., Hural J., Graham B.S., Moodie Z., Grove D., Gray G., Bekker L.G., McElrath M.J. (2011). A phase IIA randomized clinical trial of a multiclade HIV-1 DNA prime followed by a multiclade rAd5 HIV-1 vaccine boost in healthy adults (HVTN204). PLoS ONE.

[140] Chung A.W., Kumar M.P., Arnold K.B., Yu W.H., Schoen M.K., Dunphy L.J., Suscovich T.J., Frahm N., Linde C., Mahan A.E. (2015). Dissecting Polyclonal Vaccine-Induced Humoral Immunity against HIV Using Systems Serology. Cell.

[141] Haynes B.F., Gilbert P.B., McElrath M.J., Zolla-Pazner S., Tomaras G.D., Alam S.M., Evans D.T., Montefiori D.C., Karnasuta C., Sutthent R. (2012). Immune-correlates analysis of an HIV-1 vaccine efficacy trial. N. Engl. J. Med..

[142] Tomaras G.D., Ferrari G., Shen X., Alam S.M., Liao H.X., Pollara J., Bonsignori M., Moody M.A., Fong Y., Chen X. (2013). Vaccine-induced plasma IgA specific for the C1 region of the HIV-1 envelope blocks binding and effector function of IgG. Proc. Natl. Acad. Sci. USA.

[143] Yates N.L., Liao H.X., Fong Y., deCamp A., Vandergrift N.A., Williams W.T., Alam S.M., Ferrari G., Yang Z.Y., Seaton K.E. (2014). Vaccine-induced Env V1-V2 IgG3 correlates with lower HIV-1 infection risk and declines soon after vaccination. Sci. Transl. Med..

[144] Leroux-Roels G., Maes C., Clement F., van Engelenburg F., van den Dobbelsteen M., Adler M., Amacker M., Lopalco L., Bomsel M., Chalifour A. (2013). Randomized Phase I: Safety, Immunogenicity and Mucosal Antiviral Activity in Young Healthy Women Vaccinated with HIV-1 Gp41 P1 Peptide on Virosomes. PLoS ONE.

[145] Yook S.H., Oltvai Z.N., Barabasi A.L. (2004). Functional and topological characterization of protein interaction networks. Proteomics.

[146] Barabasi A.L., Oltvai Z.N. (2004). Network biology: Understanding the cell's functional organization. Nat. Rev. Genet..

[147] Barouch D.H., Alter G., Broge T., Linde C., Ackerman M.E., Brown E.P., Borducchi E.N., Smith K.M., Nkolola J.P., Liu J. (2015). Protective efficacy of adenovirus/protein vaccines against SIV challenges in rhesus monkeys. Science.

[148] Bradley T., Pollara J., Santra S., Vandergrift N., Pittala S., Bailey-Kellogg C., Shen X., Parks R., Goodman D., Eaton A. (2017). Pentavalent HIV-1 vaccine protects against simian-human immunodeficiency virus challenge. Nat. Commun..

[149] Vaccari M., Gordon S.N., Fourati S., Schifanella L., Liyanage N.P., Cameron M., Keele B.F., Shen X., Tomaras G.D., Billings E. (2016). Adjuvant-dependent innate and adaptive immune signatures of risk of SIVmac251 acquisition. Nat. Med..

